# Factors leading to excessive fatigue in nurses – a three-year follow-up study

**DOI:** 10.1186/s12912-024-02066-w

**Published:** 2024-07-01

**Authors:** Stand Hiestand, Siri Waage, Ingeborg Forthun, Ståle Pallesen, Bjørn Bjorvatn

**Affiliations:** 1 Department of Global Public Health and Primary Care, University of Bergen, PO Box 7804, Bergen, 5020 Norway; 2 Norwegian Competence Center for Sleep Disorders, Haukeland University Hospital, PO Box 1400, Bergen, 5021 Norway; 3 Department of Psychosocial Science, University of Bergen, PO Box 7807, Bergen, 5020 Norway; 4 Department of Disease Burden, Norwegian Institute of Public Health, Postboks 973 Sentrum, Bergen, 5808 Norway

**Keywords:** Fatigue, Sleep, Insomnia, Sleep Deprivation, Nurses, Work, Depression, Anxiety, Lifestyle, Shift work schedule

## Abstract

**Background:**

Global nursing shortages necessitate the identification of mitigatable factors that may reduce nursing absence and turnover. Fatigue has been shown to be associated with these issues. This study aimed to identify factors leading to development of or recovery from excessive fatigue in nurses as these can offer actionable avenues for protecting nurses against fatigue or supporting fatigue recovery.

**Methods:**

A longitudinal study among nurses randomly sampled from the Norwegian Nurse’s Organization. The Chalder Fatigue Questionnaire measured fatigue. Dichotomized scoring was used, with scores ≥ 4 considered excessive fatigue. The study included questions on shift work schedules, psychosocial work characteristics, sleep, body mass index, physical activity, caffeine, alcohol, mental health, etc. Two sets of logistic regression analysis were conducted (one for development of and one for recovery from excessive fatigue), evaluating how changes in work, lifestyle and health between baseline (2015) and follow-up (2018) affected first, odds of development of excessive fatigue and second, odds of recovery from excessive fatigue.

**Results:**

Among 1,311 included nurses, 21.6% maintained, 13.3% developed, and 18.0% recovered from excessive fatigue (2015–2018). Within work characteristics, increased psychological work demands were associated with development of excessive fatigue OR = 1.77 (CI = 1.11–2.82). Several work characteristics were associated with recovery from excessive fatigue, including decreased decision latitude (OR = 0.39; CI = 0.19–0.82) and increased coworker support (OR = 1.90; CI = 1.11–3.24). Shift work variables were not associated with fatigue outcomes. Amongst lifestyle factors, changes in sleep duration, obesity, and exercise were significant. Notably, developing inappropriate sleep duration (OR = 2.84; CI = 1.47–5.48) increased odds of developing excessive fatigue, while maintaining inappropriate sleep duration (< 6 h or > 8 h) (OR = 0.19; CI = 0.54–0.65) decreased odds of recovering. All assessed health conditions (depression, anxiety, insomnia, and shift work disorder) were related to development of (ORs 2.10–8.07) or recovery from (ORs 0.10–0.50) excessive fatigue. Depression, for example, increased odds of development of (OR = 8.07; CI = 2.35–27.66) and decreased odds of recovery (OR = 0.10; CI = 0.04–0.26) from excessive fatigue.

**Conclusions:**

Changes in lifestyle factors, health conditions, and psychosocial work factors were associated with development of and recovery from excessive fatigue. Sleep and psychosocial work factors played important roles. We found no relationship with shift work schedules.

**Supplementary Information:**

The online version contains supplementary material available at 10.1186/s12912-024-02066-w.

## Background

Fatigue lacks a singular universally agreed upon definition. It is a multidimensional concept comprising mental and/or physical components that may be categorized as acute or chronic [1]. One way to conceptualize fatigue is awareness of a reduced capacity for mental and/or physical activities [2]. Some studies choose to categorize fatigue as either physical or mental. Physical or muscular fatigue relates to reductions in ability to generate muscular force induced by neuromuscular activity, whereas mental fatigue relates to an individual’s feelings of being tired or lacking energy [1]. In real world scenarios, it may be difficult to differentiate between components of physical and mental fatigue, therefore our study uses a global fatigue score encompassing both elements.

Fatigue is a phenomenon that may overlap with, but has been demonstrated to be distinct from sleepiness [3]. Impairments in mood and cognition may be similar between sleepiness and fatigue [1]. However, sleepiness is considered to be related to the propensity to fall asleep, whereas one may experience fatigue without being more likely to doze off [1].

Fatigue’s various definitions make comparisons between studies difficult [4]. Still, fatigue is commonly reported in the general population [5], it is associated with both short and long term sick leave from work [6], and fatigue-related productivity loss is costly [7].

In a systematic review of fatigue in shift workers from 2020, the prevalence of chronic fatigue ranged from 22.7 to 50.7%, and acute fatigue from 47.8 to 69% [4]. In nurses, we have previously reported excessive fatigue, defined as scoring ≥ 4 on the dichotomized version of the Chalder Fatigue Questionnaire (also known as the Chalder Fatigue Scale), in 35.4% [8]. Fatigue in nurses may lead to patient care errors [9], absence [10], or resignation [11]. In the face of a worldwide nursing shortage [12], it is imperative to identify modifiable factors related to excessive fatigue to inform measures to retain nurses.

### Work characteristics

Some studies find clear differences in fatigue based on work schedules [13]. However, while a US survey of registered nurses found that working > 60 h per week is associated with more physical fatigue than working ≤ 40 h [14], no significant relationship between working hours and fatigue was found in South Korean nurses [15]. Among shift work factors, past studies have indicated an association between quick returns (< 11 h between shifts) and fatigue [16, 17]. Further, some studies show a significant association [17, 18] between night work and fatigue. However, others do not [8, 19]. Psychosocial work factors, such as work demands, have been associated with fatigue in Dutch and Chinese nurses [19, 20].

### Lifestyle factors

Sleep deprivation [21], body mass index (BMI) [22], physical activity [23], caffeine consumption [24], and smoking [22] have all been shown to have significant relationships with fatigue. Research on whether alcohol use causes fatigue is unclear, with one study in colorectal cancer survivors reporting less fatigue in those who increased their alcohol consumption post-treatment [25], whereas another recent study found an association between heavier alcohol consumption and self-reported tiredness [26].

### Health conditions

Depression, anxiety [19, 27] and insomnia [21] clearly associate with fatigue. Additionally, shift work disorder (a sleep disorder caused by one’s work schedule characterized by insomnia and sleepiness) has been shown to associate significantly with fatigue in cross-sectional crude analyses [8, 28].

Previous literature needs clarification, especially regarding the toll of work factors. Few studies in nurses [16, 29, 30] have examined fatigue outcomes longitudinally. Most studies are cross-sectional [8, 14, 15, 17–19, 24, 28] and therefore have been unable to conclude about directionality of relationships. Additionally, past studies have focused primarily on the development of fatigue [16, 29, 30]. However, analyzing both development of and recovery from fatigue can help identify potentially protective factors in addition to risk factors. This study therefore aims to determine if longitudinal changes in work characteristics, lifestyle factors and health conditions are associated with development of or recovery from excessive fatigue.

## Methods

### Study design and participants

The SUrvey of Shift work, Sleep and Health (SUSSH) is an ongoing annual cohort of Norwegian nurses. Between December 2008 and November 2009 initial data collection was carried out. In all, 6,000 nurses were sampled randomly from the Norwegian Nurse’s Organization using five strata (< 12 months, 1–3 years, > 3–6 years, > 6–9 years and > 9–12 years since graduation). These nurses were invited to join the study. Initial and follow-up questionnaires were mailed to participants who consented to join SUSSH, pre-paid envelopes were included for their return. Nurses who responded in SUSSH waves were included in a lottery (winners received a 500 NOK gift card). Of the initial questionnaires, 600 were returned (wrong addresses) leaving 2,059 of 5,400 nurses who responded in the first wave (38.1% response rate). In fall 2009, an additional 2,741 recent nursing graduates were invited to join the study, of which 905 agreed (33.0% response rate). Combined, these participants comprise SUSSH’s baseline cohort, a total sample of 2,964 nurses. Nurses throughout the entire country were invited throughout recruitment, albeit with about 50% of those invited working in western Norway.

**Fig. 1:**
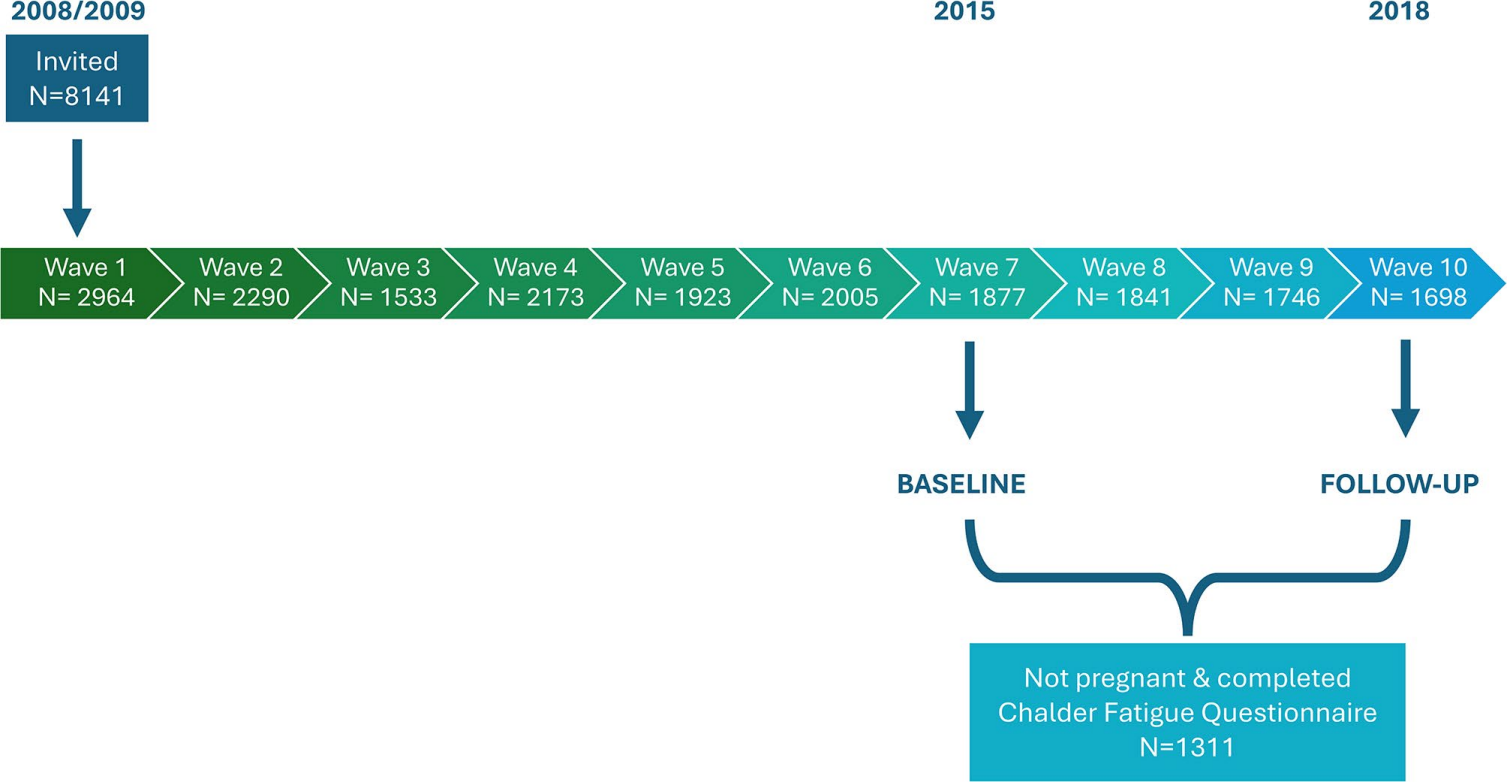
Flowchart of the survey of shiftwork sleep and health (SUSSH)

This longitudinal study has a three-year follow-up. It includes SUSSH data from waves 7 (2015) and 10 (2018). Generally, each wave of the survey contains different instruments and questions, with select measures repeated at different time intervals. For the present study, all relevant measures were included in the two waves. In wave 7, a total of 1,877 out of 2,777 eligible nurses responded (67.6% response rate). In wave 10, a total of 1,698 out of 2,774 eligible nurses responded (61.2%). Fatigue during pregnancy is prevalent and persistent [31]. As the present study focuses on work-related variables and their impact on fatigue, criteria for inclusion in the present study were not being pregnant in either wave and having completed the Chalder Fatigue Questionnaire in both waves. This left a total of 1,311 for the analytic sample (Fig. 1).

### Independent variables

#### Work characteristics

Nurses self-reported the average number of hours worked per week. Work schedule was reported as: “day only”, “evening only”, “two-shift (day and evening)”, “night only”, “three-shift (day, evening and night)” or “other schedules with night work”. Additionally, they indicated the number of nights and the number of quick returns (< 11 h between shifts) they worked the previous year. All data were collected at both time points, and calculations were made in order to classify changes in characteristics.

Based on self-reported hours, change in working hours was categorized as “no change” (at follow-up nurses worked +/-7 h per week compared to baseline), “>7 h decrease”, or “>7 h increase”. A cut-off of 7 h was used due to its similarity to a 20% decrease or increase in a full-time position in Norway. Work schedule changes were categorized as “constant day work”, “constant night work”, “starting night work” and “quitting night work”. Changes in nights and quick returns were classified as “no difference (± 10)”, “>10 decrease” or “>10 increase” as in past research [32].

Psychosocial work factors were measured using Job Content Questionnaire [33] subscales. Psychological demands (range 0–15), coworker support and decision latitude scores (range 0–18) were calculated for each timepoint. Cronbach’s alphas were 0.77 (baseline) and 0.78 (follow-up) for psychological work demands, 0.86 (baseline) and 0.84 (follow-up) for coworker support and 0.60 (baseline) and 0.57 (follow-up) for decision latitude. Variables for changes in each of these scores were created with the categories “no difference (± 2)”, “≥3 decrease” or “≥3 increase”. Scores of ± 2 are within one standard deviation of each other from baseline to follow-up.

#### Lifestyle factors

Sleep duration was self-reported in hours and minutes. Sleeping less than 6 h has been shown to associate with mortality [34]. Further, a multidisciplinary expert panel convened by The National Sleep Foundation classified sleeping ***<*** 6 h as ‘not recommended’ for either adults or young adults but indicated that 6–7 h may be appropriate [35]. Thus, while we can feel confident that sleep duration ***<*** 6 h is likely pathological, a sleep duration of 6 to 7 h may be appropriate for some individuals. We therefore classified appropriate sleep duration as 6–8 h [8, 36]. Short and long sleep durations were correspondingly categorized as < 6 and > 8 h.

Change in sleep duration was categorized as “maintaining appropriate sleep duration” (6–8 h), “maintaining inappropriate sleep duration” (< 6 or > 8 h), “recovering from inappropriate sleep duration” (< 6 or > 8 h at baseline and 6–8 h at follow-up) and “developing inappropriate sleep duration” (6–8 h at baseline and < 6 or > 8 h at follow-up).

Height and weight were self-reported. BMI was calculated as weight(kg)/height^2^ and dichotomized as obesity (BMI ≥ 30)/no obesity (BMI < 30) as in past research [37]. Change in BMI was grouped as “maintaining no obesity” (BMI < 30 both timepoints), “maintaining obesity” (BMI ≥ 30 both timepoints), “recovering from obesity” (BMI ≥ 30 at baseline and < 30 at follow-up) and “developing obesity” for the inverse.

Physical activity was self-reported as hours of sweaty exercise performed weekly (0, <1 h, 1–2 h, ≥3 h). Data was dichotomized (< 1 h/≥1 h per week) as in earlier research [37]. Change in physical activity was categorized as “maintaining ≥ 1 h/”, “maintaining < 1 h/week”, “decreasing from ≥ 1 to < 1 h per week”, or “increasing from < 1 h to ≥ 1 h per week”.

Caffeine consumption was self-reported as the average number of caffeine containing beverages consumed daily. An umbrella review of 201 meta-analyses found 3–4 cups of coffee per day was associated with optimal reduction of various health risks [38]. Because of this, and previous research [37], caffeine was dichotomized as ≥ 3/<3cups per day. Change in caffeine consumption was categorized as “maintaining ≥ 3 cups per day”, “maintaining < 3 cups per day”, “decreasing consumption from ≥ 3 to < 3 cups per day” or “increasing consumption from < 3 to ≥ 3 cups per day”.

Alcohol consumption was measured with the short form Alcohol Use Disorders Identification Test Consumption (AUDIT-C) [39], where scores of ≥ 3 for females and ≥ 4 for males are considered above cut-off (potential alcohol misuse), consumption was dichotomized accordingly. Changes in alcohol consumption were categorized as “maintaining under cut-off”, “maintaining above cut-off”, “decreasing alcohol use to below cut-off”, and “increasing alcohol use to above cut-off”.

Participants reported if they smoked daily (yes/no). Changes in smoking habit were categorized as “maintaining non-smoking”, “maintaining smoking”, “quitting smoking” and “starting smoking”.

#### Health conditions

Depression and anxiety were assessed using a validated Norwegian version [40] of the Hospital Anxiety and Depression Scale. This scale measures 7 non-vegetative depression and anxiety symptoms, respectively. Each item is scored 0–3, higher scores indicate higher symptom burdens. In accordance with prior studies [40], scoring ≥ 8 on each subscale was used as a cut-off to categorize depression or anxiety. In the present study, Cronbach’s alphas were 0.84 for anxiety (both timepoints), and 0.81 (baseline) and 0.82 (follow-up) for depression.

Insomnia symptoms were measured based on insomnia disorder inclusion criteria (Diagnostic and Statistical Manual of Mental Disorders, version 5) [41] using the Bergen Insomnia Scale [42]. The Bergen Insomnia Scale comprises six items. The first three items relate to sleep latency (taking > 30 m to fall asleep), sleep maintenance (being awake > 30 m between periods of sleep) and early awakening (waking > 30 m earlier than wished). The remaining three items relate to feeling inadequately rested by sleep, experiencing consequences at work, school or in one’s private life, and dissatisfaction with sleep. Participants reported how many days per week in the last three months they experienced each of these issues. Higher scores indicate more insomnia symptoms. Participants were considered to have insomnia if they reported ≥ 3 days per week on one or more of the first three items plus ≥ 3 days per week on one or more of the last two items. Cronbach’s alpha for the Bergen Insomnia Scale was 0.82 at both timepoints.

As in previous research [8], three questions based on the International Classification of Sleep Disorders [43] were used to assess shift work disorder: (1) “Do you have a work schedule that sometimes overlaps with the time you usually sleep?” (2) “If yes, does this cause insomnia and/or excessive sleepiness due to a reduced amount of sleep?” (3) “If yes, has this lasted for at least three months?” Participants answering “yes” to all three questions were categorized as having shift work disorder.

Changes in health conditions were categorized as “maintaining no condition”, “maintaining condition”, “recovering from condition” or “developing condition”.

### Dependent variable

Fatigue was assessed using the Norwegian version [44] of the 11-item Chalder Fatigue Questionnaire. Fatigue questions are scored with a 4-point Likert scale. A dichotomized scoring in line with Chalder Fatigue Questionnaire guidelines [45] and past research [16], was used to identify nurses with excessive fatigue. Options such as “less than usual” or “not more than usual” were grouped together (coded 0) and “more than usual” or “much more than usual” were coded 1. These scores were summed (total possible 11). Scores ≥ 4 were categorized as excessive fatigue [45]. Cronbach’s alphas were 0.90 (baseline) and 0.91 (follow-up).

### Analysis

Analyses were conducted using SPSS Statistics 28. First, continuous versions of work, lifestyle and health variables were evaluated with paired samples t-tests (Additional files 1 & 2). The two dichotomized variables (physical activity and shift work disorder) were tested with McNemar’s test (Additional files 1 & 2).

Then, two sets of crude and adjusted logistic regressions were performed. The first set of regressions were performed on nurses without excessive fatigue at baseline (*n* = 792) with fatigue status at follow-up as the dependent variable (maintaining no fatigue = 0 and developing fatigue = 1). The second set of logistic regressions were performed on nurses who reported excessive fatigue at baseline (*n* = 519) with fatigue status at follow-up as the dependent variable (maintaining fatigue = 0 and recovering from fatigue = 1). For both sets of regressions separate analyses were conducted to determine the effects of changes in work characteristics, lifestyle factors and health conditions from baseline to follow-up on fatigue status. The independent variables analyzed include:

Work characteristics:

1. Work schedule.
2. Working hours.
3. Numbers of nights.
4. Number of quick returns.
5. Psychological work demands.
6. Coworker support score.
7. Decision latitude score.

Lifestyle factors:

1. Sleep duration.
2. Obesity.
3. Physical activity.
4. Caffeine consumption.
5. Alcohol consumption.
6. Smoking habits.

Health conditions:

1. Depression.
2. Anxiety.
3. Insomnia.
4. Shift work disorder.

Adjusted models included possible confounding variables: sex (female/male), age (continuous), baseline marital status (partnered, no/yes) and children at home at baseline (no/yes). All variables are included in Tables 1 and 2 (descriptive characteristics at baseline and changes in variables from baseline to follow-up). Thereafter variables with no significant relationships with excessive fatigue are included only in additional files. Odds ratios (OR) including 95% confidence intervals (CI) are reported. Regressions used complete-case analysis (missing data was not included).

The types of health problems associated with long sleep duration may differ from those of short sleepers. Therefore, sensitivity analyses were run on nurses developing or recovering from excessive fatigue, excluding nurses sleeping > 8 h (Additional file 8). The same changes in sleep duration were significant and associated in the same directions. Point estimates for odds ratios were very slightly higher for development of and somewhat lower for recovery from excessive fatigue when excluding sleeping > 8 h, but the sample sizes were smaller, and CIs were generally wider.

Age (the only included continuous variable) was linearly related to the logit of the dependent variable in all models according to the Box-Tidwell procedure. Multicollinearity was tested but not found, variance inflation factor scores were between 1.02 and 1.42.

## Results

The group consisted of 1,172 females (89.8%) and 133 males (10.2%). The average age of nurses was 39.2 (8.6), and the majority (*n* = 1018, 78.1%) were married or cohabitating at baseline. Nearly 40% reported excessive fatigue at baseline (Table 1).

**Table 1:** Work characteristics, lifestyle factors, health conditions, and fatigue in 1,311 participating nurses at baseline

**Work characteristics**	
Work schedule (*n* = 1106)	
Day only (*n* = 259)	23.4%
Evening only (*n* = 1)	0.1%
Two-shift (day and evening) (*n* = 353)	31.9%
Night only (*n* = 74)	6.7%
Three-shift (day, evening and night) (*n* = 376)	34.0%
Other schedules with night work (*n* = 43)	3.9%
Working hours at baseline (*n* = 1191) mean (SD)	34.0 (6.7)
Number of nights last year (*n* = 1205)	20.0 (34.1)
Number of quick returns last year (*n* = 1194)	29.2 (35.5)
Psychological work demands^1^ (*n* = 1210), mean (SD)	9.2 (2.7)
Coworker support^1^ (*n* = 1206), mean (SD)	13.6 (2.9)
Decision latitude^1^ (*n* = 1206), mean (SD)	11.6 (2.4)
**Lifestyle factors**	
Sleep duration (*n* = 1302)	
Appropriate sleep duration (6–8 h) (*n* = 1167)	89.6%
Short sleep duration (< 6 h) (*n* = 100)	7.7%
Long sleep duration (> 8 h) (*n* = 35)	2.7%
BMI (*n* = 1161) mean (SD)	25.2 (4.7)
No obesity (*n* = 1014) (BMI < 30)	87.3%
Obesity (*n* = 147) (BMI ≥ 30)	12.7%
Physical activity *n* = 1273	
≥1 h/week (*n* = 785)	61.7%
<1 h/week (*n* = 488)	38.3%
Caffeine consumption (*n* = 1309)	
≥3 cups per day (*n* = 880)	67.2%
<3 cups per day (*n* = 429)	32.8%
Alcohol consumption^2^ (*n* = 1191)	
Not above cut-off for possible alcohol misuse (*n* = 526)	44.2%
Above cut-off for possible alcohol misuse (*n* = 665)	55.8%
Smoking habits (*n* = 1309)	
No smoking (*n* = 1207)	92.2%
Daily smoking (*n* = 102)	7.8%
**Health Conditions**	
Depression^3^ (*n* = 1301)	
No depression (*n* = 1177)	90.5%
Depression (*n* = 124)	9.5%
Anxiety^3^ (*n* = 1297)	
No anxiety (*n* = 980)	75.6%
Anxiety (*n* = 317)	24.4%
Insomnia^4^ (*n* = 1311)	
No insomnia (*n* = 885)	67.5%
Insomnia (*n* = 426)	32.5%
Shift work disorder^5^ (*n* = 1302)	
No shift work disorder (*n* = 919)	70.6%
Shift work disorder (*n* = 383)	29.4%
*Excessive fatigue*^6^ (*n* = 1311)	
No excessive fatigue (*n* = 792)	60.4%
Excessive fatigue (*n* = 519)	39.6%

^1^Measured with subscales of the Job Content Questionnaire, psychological demands score ranges 0–15, coworker support and decision latitude scores range 0–18. ^2^Measured with the short form Alcohol Use Disorders Identification Test Consumption (AUDIT-C), scores of ≥ 3 for females and ≥ 4 for males indicate above cut-off use (potential alcohol misuse). ^3^Measured with the Hospital Anxiety and Depression Scale (range for each 0–21). Scores ≥ 8 for either anxiety subscale or depression subscale defined as having anxiety or depression respectively. ^4^Measured with the Bergen Insomnia Scale (range 0–42). Insomnia categorization based on DSM-5 criteria. ^5^Shift work disorder, assessed with 3 questions adhering to International Classification of Sleep Disorders ed. 3. ^6^Chalder Fatigue Questionnaire dichotomized sum score ≥ 4 considered excessive fatigue

**Fig. 2:**
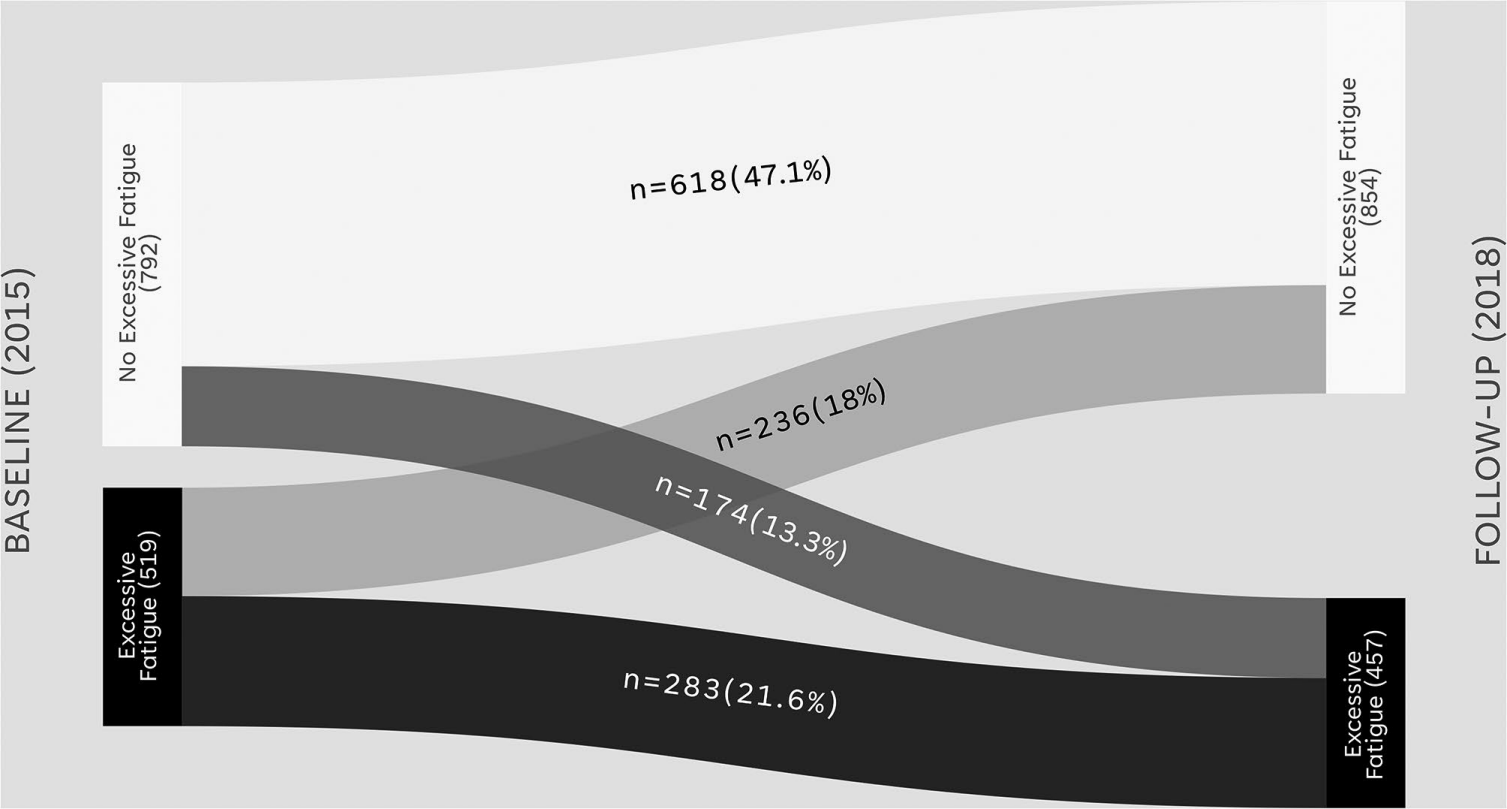
Changes in excessive fatigue in 1311 participating nurses between baseline and follow-up. Legend: Chalder Fatigue Questionnaire dichotomized sum score ≥ 4 considered excessive fatigue. Figure made with SankeyMATIC

Nearly 35% of nurses either maintained or developed excessive fatigue, while roughly 65% maintained no excessive fatigue or recovered from it (Fig. 2). Over 80% had no change in their work hours (Table 2). Many factors either remained stable or moved to the ‘healthy’ category for each variable. For example, nearly 60% of nurses maintained or increased physical activity, while 90% maintained appropriate or recovered from inappropriate sleep duration (Table 2).

**Table 2:** Changes in work characteristics, lifestyle factors, and health conditions in 1,311 participating nurses between baseline and follow-up

**Changes in work characteristics**	
Change in work schedule (*n* = 991)	
Constant day work (*n* = 485)	48.9%
Constant night work (n = 352)	35.5%
Quitting night work (*n* = 102)	10.3%
Starting night work (n = 52)	5.2%
Change in working hours (*n* = 1130)	
No change (+/-7 h) (*n* = 930)	82.3%
>7 h decrease (*n* = 97)	8.6%
>7 h increase (*n* = 103)	9.1%
Change in numbers of nights (*n* = 1147)	
No difference (± 10) (*n* = 802)	69.9%
>10 decrease (*n* = 204)	17.8%
>10 increase (*n* = 141)	12.3%
Change in number of quick returns^1^ worked last year (*n* =1130)	
No difference (± 10) (*n* = 635)	56.2%
>10 decrease (*n* = 289)	25.6%
>10 increase (*n* = 206)	18.2%
Change in psychological work demands score^2^ (*n* =1157)	
No difference (± 2) (*n* = 798)	69.0%
≥3 decrease (*n* = 174)	15.0%
≥3 increase (*n* = 185)	16.0%
Change in coworker support score^2^ (*n* =1148)	
No difference (± 2) (*n* = 704)	61.3%
≥3 decrease (*n* = 268)	23.3%
≥3 increase (*n* = 176)	15.3%
Change in decision latitude score^2^ (*n* = 1152)	
No difference (± 2) (*n* = 873)	75.8%
≥3 decrease (*n* = 122)	10.6%
≥3 increase (*n* = 157)	13.6%
**Changes in lifestyle factors**	
Change in sleep duration (*n* = 1300)	
Maintaining appropriate sleep duration (6–8 h) (*n* = 1077)	82.8%
Maintaining inappropriate sleep duration (< 6 h or > 8 h) (*n* = 42)	3.2%
Recovering from inappropriate sleep duration^3^ (*n* = 93)	7.2%
Developing inappropriate sleep duration^4^ (*n* = 88)	6.8%
Change in obesity (*n* = 1053)	
Maintaining no obesity (BMI < 30) (*n* = 885)	84.0%
Maintaining obesity (BMI ≥ 30) (*n* = 108)	10.3%
Recovering from obesity^5^ (*n* = 26)	2.5%
Developing obesity^6^ (*n* = 34)	3.2%
Change in physical activity (*n* = 1235)	
Maintaining ≥ 1 h/week (*n* = 579)	46.9%
Maintaining < 1 h/week (*n* = 310)	25.1%
Decreasing from ≥ 1 to < 1 h/week (*n* = 191)	15.5%
Increasing from < 1 h to ≥ 1 h/week (*n* = 155)	12.6%
Change in caffeine consumption from (*n* = 1305)	
Maintaining ≥ 3 cups per day (*n* = 792)	60.7%
Maintaining < 3 cups per day (*n* = 291)	22.3%
Decreasing consumption from ≥ 3 to < 3 cups per day (*n* = 87)	6.7%
Increasing consumption from < 3 to ≥ 3 cups per day (*n* = 135)	10.3%
Change in alcohol consumption^7^ (*n* = 1154)	
Maintaining under cut-off alcohol use (*n* = 382)	33.1%
Maintaining above cut-off alcohol use (*n* = 545)	47.2%
Decreasing alcohol use to below cut-off (*n* = 108)	9.4%
Increasing alcohol use to above cut-off (*n* = 119)	10.3%
Change in smoking habits (*n* = 1307)	
Maintaining non-smoking (*n* = 1170)	89.5%
Maintaining smoking (*n* = 59)	4.5%
Quitting smoking (*n* = 43)	3.3%
Starting smoking (*n* = 35)	2.7%
**Changes in health conditions**	
Change in depression^8^ (*n* = 1290)	
Maintaining no depression (*n* = 1086)	84.2%
Maintaining depression (*n* = 59)	4.6%
Recovering from depression (*n* = 63)	4.9%
Developing depression (*n* = 82)	6.4%
Change in anxiety^8^ (*n* = 1285)	
Maintaining no anxiety (*n* = 830)	64.6%
Maintaining anxiety (*n* = 186)	14.5%
Recovering from anxiety (*n* = 127)	9.9%
Developing anxiety (*n* = 142)	11.1%
Change in insomnia^9^ (*n* = 1311)	
Maintaining no insomnia (*n* = 721)	55.0%
Maintaining insomnia (*n* = 249)	19.0%
Recovering from insomnia (*n* = 177)	13.5%
Developing insomnia (*n* = 164)	12.5%
Change in shift work disorder^10^ (*n* = 1292)	
Maintaining no shift work disorder (*n* = 747)	57.8%
Maintaining shift work disorder (*n* = 228)	17.6%
Recovering from shift work disorder (*n* = 152)	11.8%
Developing shift work disorder (*n* = 165)	12.8%
Change in excessive fatigue^11^ (*n* = 1311)	
Maintaining no excessive fatigue (*n* = 618)	47.1%
Maintaining excessive fatigue (*n* = 283)	21.6%
Recovering from excessive fatigue (*n* = 236)	18.0%
Developing excessive fatigue (*n* = 174)	13.3%

^1^<11 h between consecutive work shifts. ^2^Measured with subscales of the Job Content Questionnaire, psychological demands score ranges 0–15, coworker support and decision latitude scores range 0–18. “No difference” means a participant’s score in 2015 is within one standard deviation of their score for 2018, ≥ 3 decrease in score is equivalent to at least one standard deviation increase in score from 2015 to 2018 and ≥ 3 decrease in score is equivalent to at least one standard deviation decrease in score from 2015 to 2018. ^3^Sleeping <6 h or > 8 h in 2015 and 6–8 h in 2018. ^4^Sleeping 6-8 h in 2015 and sleeping < 6 h or > 8 h in 2018. ^5^Moving from BMI ≥ 30 in 2015 to BMI < 30 in 2018. ^6^Moving from BMI < 30 in 2015 to BMI ≥ 30 in 2018. ^7^Measured with the short form Alcohol Use Disorders Identification Test Consumption (AUDIT-C), scores of ≥3 for females and ≥4 for males indicate above cut-off use (potential alcohol misuse). ^8^Measured with the Hospital Anxiety and Depression Scale (range for each 0–21). Scores ≥ 8 for either anxiety subscale or depression subscale defined as having anxiety or depression respectively. ^9^Measured with the Bergen Insomnia Scale (range 0–42). Insomnia categorization based on DSM-5 criteria. ^10^Shift work disorder, assessed with 3 questions adhering to International Classification of Sleep Disorders ed. 3. ^11^Chalder Fatigue Questionnaire dichotomized sum score ≥ 4 considered excessive fatigue

### Developing excessive fatigue

#### Work characteristics

A total of 792 nurses who reported no excessive fatigue at baseline were included in the analysis of factors which led to development of excessive fatigue. Of these, 174 developed excessive fatigue. Nurses who decreased working hours (aOR = 2.29, CI = 1.31–4.02) or had increased psychological work demands (aOR = 1.77, CI = 1.11–2.82) had significantly increased odds of developing excessive fatigue by follow-up. Other work characteristics (Table 3 & Additional file 3) were not significant.

**Table 3:** Crude and adjusted logistic regression analyses with developing excessive fatigue between baseline and follow-up as the dependent variable

	OR (95% CI)^a^	OR (95% CI)^b^
Sex (*n* = 789)		
Female (*n* = 703)	1.00	
Male (*n* = 86)	1.64 (1.00–2.69)	
Age (*n* = 791)	0.98 (0.96–1.00)	
Marital status (*n* = 782–786)		
Married/cohabitating no (*n* = 174–175)	1.00	
Married cohabitating yes (*n* = 608–611)	1.01 (0.67–1.52)	
Children at home (*n* = 778–782)		
Children living at home no (*n* = 247–248)	1.00	
Children living at home yes (*n* = 531–534)	0.89 (0.62–1.28)	
Change in working hours (*n* = 693–704)		
No change (+/-7 h) (*n* = 577–588)	1.00	1.00
>7 h decrease (*n* = 63)	**2.18** (1.25–3.81)	**2.29** (1.31–4.02)
>7 h increase (*n* = 53)	1.32 (0.69–2.55)	1.31 (0.68–2.56)
Change in psychological work demands score^1^ (*n* = 708–721)		
No difference (± 2) (*n* = 493–502)	1.00	1.00
≥3 decrease (*n* = 101–102)	0.97 (0.56–1.67)	0.89 (0.51–1.56)
≥3 increase (*n* = 114–117)	**1.88** (1.20–2.95)	**1.77** (1.11–2.82)
Change in coworker support score^1^ (*n* = 702–716)		
No difference (± 2) (*n* = 444–453)	1.00	1.00
≥3 decrease (*n* = 160–163)	1.16 (0.76–1.78)	1.16 (0.75–1.80)
≥3 increase (*n* = 98–100)	0.78 (0.44–1.38)	0.77 (0.43–1.39)
Change in decision latitude score^1^ (*n* = 705–719)		
No difference (± 2) (*n* = 542–552)	1.00	1.00
≥3 decrease (*n* = 78–81)	1.06 (0.61–1.86)	0.91 (0.50–1.66)
≥3 increase (*n* = 85–86)	0.92 (0.52–1.62)	0.94 (0.53–1.67)
Change in sleep duration (*n* = 769–783)		
Maintaining appropriate sleep duration (6–8 h) (*n* = 664–677)	1.00	1.00
Maintaining inappropriate sleep duration (< 6 or > 8 h) (*n* = 21)	2.03 (0.80–5.12)	2.11 (0.83–5.40)
Recovering from inappropriate sleep duration^2^ (*n* = 43)	**1.96** (1.01–3.81)	**2.10** (1.07–4.12)
Developing inappropriate sleep duration^3^ (*n* = 41–42)	**3.04** (1.60–5.76)	**2.84** (1.47– 5.48)
Change in Obesity (*n* = 623–636)		
Maintaining no obesity (BMI < 30) (*n* = 535–546)	1.00	1.00
Maintaining obesity (BMI ≥ 30) (*n* = 58–59)	1.14 (0.61–2.15)	1.07 (0.55–2.06)
Recovering from obesity^4^ (*n* = 15)	1.83 (0.62–5.47)	1.95 (0.64–5.95)
Developing obesity^5^ (*n* = 15–16)	**2.85** (1.04–7.82)	2.42 (0.83–7.04)
Change in physical activity (*n* = 731–745)		
Maintaining ≥ 1 h/week (*n* = 398–404)	1.00	1.00
Maintaining < 1 h/week (*n* = 145–149)	1.15 (0.73–1.82)	1.21 (0.76–1.94)
Decreasing from ≥ 1 to < 1 h/week (*n* = 109–112)	**1.77** (1.10–2.83)	**1.68** (1.03–2.74)
Increasing from < 1 h to ≥ 1 h/week (*n* = 79–80)	1.01 (0.56–1.85)	1.11 (0.60–2.03)
Change in depression^6^ (*n* = 769–784)		
Maintaining no depression (*n* = 716–730)	1.00	1.00
Maintaining depression (*n* = 12)	**8.43** (2.50–28.39)	**8.07** (2.35–27.66)
Recovering from depression (*n* = 11)	1.58 (0.41–6.03)	1.65 (0.43–6.41)
Developing depression (*n* = 30–31)	**7.66** (3.59–16.36)	**6.96** (3.21–15.06)
Change in anxiety^6^ (*n* = 764–779)		
Maintaining no anxiety (*n* = 591–604)	1.00	1.00
Maintaining anxiety (*n* = 51–52)	**4.56** (2.54–8.17)	**4.41** (2.42–8.04)
Recovering from anxiety (*n* = 50)	0.94 (0.43–2.06)	1.03 (0.47–2.27)
Developing anxiety (*n* = 72–73)	**5.06** (3.05–8.39)	**5.04** (3.00–8.44)
Change in insomnia^7^ (*n* = 777–792)		
Maintaining no insomnia (*n* = 520–529)	1.00	1.00
Maintaining insomnia (*n* = 86–88)	**2.65** (1.62–4.34)	**3.34** (1.99–5.60)
Recovering from insomnia (*n* = 70–71)	1.09 (0.58–2.08)	1.18 (0.61–2.26)
Developing insomnia (*n* = 101–104)	**3.05** (1.93–4.81)	**3.04** (1.89–4.89)
Change in shift work disorder^8^ (*n* = 767–780)		
Maintaining no shift work disorder (*n* = 501–508)	1.00	1.00
Maintaining shift work disorder (*n* = 94–97)	**1.88** (1.15–3.06)	**2.10** (1.27–3.47)
Recovering from shift work disorder (*n* = 79–81)	0.92 (0.50–1.71)	1.01 (0.54–1.89)
Developing shift work disorder (*n* = 93–94)	**2.73** (1.71–4.38)	**2.64** (1.63–4.29)

^a^Separate crude logistic regression analyses for each independent variable. ^b^Separate logistic regression analyses for each independent variable adjusted for sex, age, and marital status and children at home at baseline. ^1^Measured with subscales of the Job Content Questionnaire, psychological demands score ranges 0–15, coworker support and decision latitude scores range 0–18. “No difference” means a participant’s score in 2015 is within one standard deviation of their score for 2018, ≥ 3 decrease in score is equivalent to at least one standard deviation increase in score from 2015 to 2018 and ≥ 3 decrease in score is equivalent to at least one standard deviation decrease in score from 2015 to 2018. ^2^Sleeping <6 h or > 8 h in 2015 and 6–8 h in 2018. ^3^Sleeping 6-8 h in 2015 and sleeping < 6 h or > 8 h in 2018. ^4^Moving from BMI ≥ 30 in 2015 to BMI < 30 in 2018. ^5^Moving from BMI < 30 in 2015 to BMI ≥ 30 in 2018. ^6^Measured with the Hospital Anxiety and Depression Scale (range for each 0–21). Scores ≥ 8 for either anxiety subscale or depression subscale defined as having anxiety or depression respectively. ^7^Measured with the Bergen Insomnia Scale (range 0–42). Insomnia categorization based on DSM-5 criteria. ^8^Shift work disorder, assessed with 3 questions adhering to International Classification of Sleep Disorders ed. 3. Significant findings are shown in **bold**. Chalder Fatigue Questionnaire dichotomized sum score ≥ 4 considered excessive fatigue. Variable used for regression: excessive fatigue at both time points vs. fatigue develops

#### Lifestyle factors

Recovering from (aOR = 2.10, CI = 1.07–4.12) or developing (aOR = 2.84, CI = 1.47– 5.48) inappropriate sleep duration and decreasing physical activity (aOR = 1.68, CI = 1.03–2.74) increased odds of developing excessive fatigue. Developing obesity was not significantly associated in the fully adjusted model.

#### Health conditions

Maintaining or developing depression, anxiety, insomnia or shift work disorder all increased the odds of developing excessive fatigue, ranging from aOR = 2.10 CI = 1.27–3.47) (maintaining shift work disorder) to aOR = 8.07, CI = 2.35–27.66 (maintaining depression) (Table 3).

### Recovering from excessive fatigue

#### Work characteristics

A total of 519 nurses who had excessive fatigue at baseline were included in the analysis of factors which led to recovery from excessive fatigue, 236 of these recovered from fatigue. Changes in psychosocial work factors, but not work hours (Table 4) or shift work schedule, number of night shifts or number of quick returns (Additional file 4), were significantly associated with recovery from excessive fatigue. A decrease of psychological work demands (adjusted odds ratio (aOR) = 1.76, CI = 1.02–3.05) increased odds of recovery nearly two-fold, while an increase of psychological work demands decreased odds of recovery (aOR = 0.53, CI = 0.30–0.93). Increasing coworker support increased odds of recovery (aOR = 1.90, CI = 1.11–3.24), while decreasing decision latitude decreased odds of recovery (aOR = 0.39, CI = 0.19–0.82).

**Table 4:** Crude and adjusted logistic regression analyses with recovering from excessive fatigue between baseline and follow-up as the dependent variable

	OR (95% CI)^a^	OR (95% CI)^b^	
Sex (*n* = 516)			
Female (*n* = 469)	1.00		
Male (*n* = 47)	0.88 (0.48–1.62)		
Age (*n* = 517)	**0.98** (0.96–1.00)		
Marital status (*n* = 513–517)			
Married/cohabitating no (*n* = 108–110)	1.00		
Married cohabitating yes (*n* = 405–407)	0.96 (0.63–1.47)		
Children at home (*n* = 511–515)			
Children living at home no (*n* = 148–150)	1.00		
Children living at home yes (*n* = 363–365)	1.27 (0.86–1.86)		
Change in working hours (*n* = 420–426)			
No change (+/-7 h) (*n* = 336–342)	1.00	1.00	
>7 h decrease (*n* = 34)	0.66 (0.32–1.37)	0.64 (0.30–1.35)	
>7 h increase (*n* = 50)	1.67 (0.91–3.04)	1.56 (0.85–2.87)	
Change in psychological work demands score^1^ (*n* = 430–436)			
No difference (± 2) (*n* = 293–296)	1.00	1.00	
≥3 decrease (*n* = 70–72)	**2.03** (1.19–3.45)	**1.76** (1.02–3.05)	
≥3 increase (*n* = 67–68)	**0.55** (0.31–0.96)	**0.53** (0.30–0.93)	
Change in coworker support score^1^ (*n* = 426–432)			
No difference (± 2) (*n* = 247–251)	1.00	1.00	
≥3 decrease (*n* = 103–105)	0.95 (0.60–1.51)	0.91 (0.57–1.46)	
≥3 increase (*n* = 76)	**1.87** (1.11–3.16)	**1.90** (1.11–3.24)	
Change in decision latitude score^1^ (*n* = 427–433)			
No difference (± 2) (*n* = 316–321)	1.00	1.00	
≥3 decrease (*n* = 40–41)	**0.40** (0.20–0.83)	**0.39** (0.19–0.82)	
≥3 increase (*n* = 71)	1.50 (0.89–2.52)	1.53 (0.90–2.60)	
Change in sleep duration (*n* = 511–517)			
Maintaining appropriate sleep duration (6–8 h sleep) (*n* = 395–400)	1.00	1.00	
Maintaining inappropriate sleep duration (< 6 h or > 8 h) (*n* = 21)	**0.17** (0.05–0.59)	**0.19** (0.54–0.65)	
Recovering from inappropriate sleep duration^2^ (*n* = 50)	0.80 (0.44–1.45)	0.77 (0.42–1.40)	
Developing inappropriate sleep duration^3^ (*n* = 45–46)	**0.36** (0.18–0.72)	**0.37** (0.18–0.74)	
Change in obesity (*n* = 414–417)			
Maintaining no obesity (BMI < 30) (*n* = 333–339)	1.00	1.00	
Maintaining obesity (BMI ≥ 30) (*n* = 48–49)	**0.40** (0.21–0.79)	**0.37** (0.18–0.73)	
Recovering from obesity^4^ (*n* = 11)	1.34 (0.40–4.48)	1.38 (0.41–4.70)	
Developing obesity^5^ (*n* = 18)	**0.32** (0.10–0.99)	**0.27** (0.09–0.86)	
Change in physical activity (*n* = 482–490)			
Maintaining ≥ 1 h/week (*n* = 172–175)	1.00	1.00	
Maintaining < 1 h/week (*n* = 158–161)	0.71 (0.46–1.09)	0.67 (0.43–1.04)	
Decreasing from ≥ 1 to < 1 h/week (*n* = 79)	**0.55** (0.32–0.95)	**0.53** (0.31–0.92)	
Increasing from < 1 h to ≥ 1 h/week (*n* = 73–75)	1.02 (0.60–1.76)	0.99 (0.57–1.73)	
Change in depression^6^ (*n* = 498–506)			
Maintaining no depression (*n* = 350–356)	1.00	1.00	
Maintaining depression (*n* = 46–47)	**0.13** (0.05–0.31)	**0.10** (0.04–0.26)	
Recovering from depression (*n* = 51–52)	0.80 (0.45–1.43)	0.82 (0.45–1.48)	
Developing depression (*n* = 51)	**0.19** (0.09–0.39)	**0.17** (0.08–0.37)	
Change in anxiety^6^ (*n* = 498–506)			
Maintaining no anxiety (*n* = 222–226)	1.00	1.00	
Maintaining anxiety (*n* = 131–134)	**0.28** (0.18–0.45)	**0.25** (0.15–0.40)	
Recovering from anxiety (*n* = 77)	0.73 (0.44–1.23)	0.76 (0.45–1.30)	
Developing anxiety (*n* = 68–69)	**0.31** (0.18–0.56)	**0.31** (0.17–0.56)	
Change in insomnia^7^ (*n* = 511–519)			
Maintaining no insomnia (*n* = 191–192)	1.00	1.00	
Maintaining insomnia (*n* = 156–161)	**0.44** (0.28–0.68)	**0.45** (0.29–0.71)	
Recovering from insomnia (*n* = 106)	1.40 (0.87–2.27)	1.41 (0.87–2.30)	
Developing insomnia (*n* = 58–60)	**0.46** (0.25–0.84)	**0.44** (0.24–0.82)	
Change in shift work disorder^8^ (*n* =504–512)			
Maintaining no shift work disorder (*n* = 237–239)	1.00	1.00	
Maintaining shift work disorder (*n* = 129–131)	**0.50** (0.32–0.78)	**0.50** (0.32–0.78)	
Recovering from shift work disorder (*n* = 69–71)	0.86 (0.51–1.46)	0.90 (0.52–1.55)	
Developing shift work disorder (*n* = 69–71)	0.91 (0.54–1.55)	0.91 (0.53–1.57)	

^a^Separate crude logistic regression analyses for each independent variable. ^b^Separate logistic regression analyses for each independent variable adjusted for sex, age, and marital status and children at home at baseline. ^1^Measured with subscales of the Job Content Questionnaire, psychological demands score ranges 0–15, coworker support and decision latitude scores range 0–18. “No difference” means a participant’s score in 2015 is within one standard deviation of their score for 2018, ≥ 3 decrease in score is equivalent to at least one standard deviation increase in score from 2015 to 2018 and ≥ 3 decrease in score is equivalent to at least one standard deviation decrease in score from 2015 to 2018. ^2^Sleeping <6 h or > 8 h in 2015 and 6–8 h in 2018. ^3^Sleeping 6-8 h in 2015 and sleeping < 6 h or > 8 h in 2018. ^4^Moving from BMI ≥ 30 in 2015 to BMI < 30 in 2018. ^5^Moving from BMI < 30 in 2015 to BMI ≥ 30 in 2018. ^6^Measured with the Hospital Anxiety and Depression Scale (range for each 0–21). Scores ≥ 8 for either anxiety subscale or depression subscale defined as having anxiety or depression respectively. ^7^Measured with the Bergen Insomnia Scale (range 0–42). Insomnia categorization based on DSM-5 criteria. ^8^Shift work disorder, assessed with 3 questions adhering to International Classification of Sleep Disorders ed. 3. Significant findings are shown in **bold**. Chalder Fatigue Questionnaire dichotomized sum score ≥ 4 considered excessive fatigue. Variable used for regression: excessive fatigue at both time points vs. fatigue disappears

#### Lifestyle factors

Changes in sleep duration, obesity, and physical activity had significant associations with recovery from excessive fatigue (Table 4), while changes in caffeine, alcohol and smoking did not (Additional file 4). Maintaining inappropriate sleep duration decreased odds of recovery (aOR = 0.19, CI = 0.54–0.65), as did maintaining (aOR = 0.37, CI = 0.18–0.73) or developing obesity (aOR = 0.27, CI = 0.09–0.86) and decreasing physical activity (aOR = 0.53, CI = 0.31–0.92).

#### Health conditions

Maintaining or developing depression, anxiety or insomnia lowered odds of recovery from excessive fatigue (Table 4). Adjusted ORs ranged from 0.10, CI = 0.04–0.26 (maintaining depression), to 0.45, CI = 0.29–0.71 (maintaining insomnia). Nurses who maintained shift work disorder (aOR = 0.50, CI = 0.32–0.78) also had lower odds of recovery.

## Discussion

Our longitudinal three-year follow-up study found that psychosocial work factors, several lifestyle factors, and depression, anxiety, insomnia and shift work disorder were associated with developing or recovering from excessive fatigue. Shift work schedule variables were not significantly associated. This study builds on cross-sectional findings [8, 14, 15, 17–19, 24, 28] and adds to previous longitudinal nursing literature [16, 29, 30].

Our study design offers some added assurances regarding directionality between study variables and excessive fatigue. It accounts for fatigue at baseline, as nurses with fatigue at baseline were analyzed separately from those who were fatigue free at baseline. Further, in situations where maintaining or developing certain work characteristics, lifestyle factors or health conditions over time both increased odds of developing fatigue and decreased the odds of recovery, there can be increased confidence that the variable impacts fatigue. One example of this in our data is depression. Developing depression both increased the odds of developing excessive fatigue and reduced the odds of recovering from it. Here, because we analyzed the development of depression, we controlled for depression at baseline in addition to fatigue. This supports the development of depression preceding fatigue, although it does not eliminate the possibility of a bi-directional relationship. Other factors, including anxiety, insomnia and shift work disorder, also both increased the odds of developing fatigue and decreased the odds of recovering from it. The consistency in these findings brings increased confidence concerning the impacts of depression, anxiety, insomnia and shift work disorder on excessive fatigue.

The present study aimed to identify potential protective factors in addition to risk factors. The majority of our significant findings for recovery from fatigue showed decreased odds of recovery (ORs ranging from 0.10 to 0.53). This suggests that these variables are real risk factors for excessive fatigue. However, we did identify two psychosocial factors (decreased psychological work demands and increased coworker social support) that were significantly associated with recovery from excessive fatigue. These may therefore offer a potential protective or even recuperative effect in terms of fatigue.

To our knowledge, this study is the first to longitudinally examine the effects of changes in work characteristics, lifestyle factors and health conditions on development of and recovery from excessive fatigue in nurses.

### Work characteristics

Decreasing working hours by > 7 h increased odds of developing excessive fatigue, which initially appears to conflict with prior research indicating that working more hours is associated with fatigue [14]. However, nurses may have reduced their working hours in response to fatigue. Nurses who reduced their hours by > 7 h between baseline and follow-up worked more than the other groups at baseline. Those who reduced their hours worked an average of 37.2 (8.9) hours per week, while those who maintained their schedule worked 34.8 (5.1) and those who increased their hours by > 7 h worked 24.5 (8.3). We cannot, however, conclude that nurses reduced their work in response to fatigue because we cannot pinpoint exactly when nurses reduced their hours in relation to the onset of excessive fatigue (fatigue data were available only in 2015 and 2018).

Our findings on the importance of psychosocial work factors were in line with past longitudinal work in a nursing context [29]. Increasing psychosocial work burdens, e.g., increased psychological work demands or decreased decision latitude, decreased odds of recovery from excessive fatigue. Conversely, decreasing burdens, e.g., decreased psychological work demands or increased coworker support, increased odds of recovery. Our findings bolster past cross-sectional [19] and longitudinal research [20] showing that work demands increase fatigue, and decision latitude and coworker support protects against fatigue. The fact that psychosocial work factors were the only factors within the study to be associated with higher odds of recovery from excessive fatigue points to the psychosocial working environment’s key role in mitigating fatigue amongst employees.

Contrasting with past longitudinal [16] and cross-sectional studies [17], we found no significant relationships between quick returns and excessive fatigue. As the SUSSH cohort began in 2008/09, participants in 2015 had a minimum of 6 years of experience working as nurses. Thus, the 2015 and 2018 participants were already experienced nurses. Experienced nurses may develop effective coping mechanisms to combat fatigue related to shift work schedules, potentially weakening associations between shift work schedule variables and fatigue. Additionally, shift workers in past studies who left their positions reported higher fatigue prior to quitting shift work than those who retained their positions [13]. Fatigued shift working nurses may have changed schedules before 2015, and the remaining group may thus be quite tolerant to shift work. This selection bias may also explain the lack of significant relationships between working nights and fatigue in the current study, in contrast to prior cross-sectional studies [17, 18].

If there are nurses remaining in our cohort who are not shift work tolerant, these may be those reporting shift work disorder. Changes in shift work disorder did show significant relationships with fatigue development and recovery. However, even considering there were nurses within the cohort reporting shift work disorder, changes in the other shift work variables did not show significant relationships with fatigue outcomes. This may indicate that, if the shift worker is not experiencing direct negative consequences such as shift work disorder, their differences in reporting fatigue may be negligible compared to those not working shifts.

### Lifestyle factors

In line with past research, sleep duration [21], BMI [22], and physical activity [23] were significantly associated with excessive fatigue. However, unlike prior research we did not find relationships between excessive fatigue and caffeine [24], alcohol [26] or smoking [22]. This may be due to actual relationships or selection bias, healthy worker effect, or insufficient sample size, especially in terms of smokers.

Our results strengthen past cross-sectional research indicating the importance of sleep within nurse fatigue [8, 14, 18, 19, 24]. Earlier studies show that insufficient sleep [8, 14, 21] is associated with fatigue. We found that developing inappropriate sleep duration increased odds of developing excessive fatigue and maintaining or developing inappropriate sleep duration decreased odds of recovery from excessive fatigue. Maintaining inappropriate sleep duration had an aOR of 0.19 for recovery from excessive fatigue, which translates to 5x the odds of *not* recovering from fatigue, emphasizing sleep’s important role in fatigue. However, recovering from inappropriate sleep duration increased odds of developing excessive fatigue. This may be due to being unable to determine exactly when excessive fatigue developed versus when sleep duration changed. Nurses who recovered from inappropriate sleep duration may have begun to sleep more in response to feeling fatigued.

The remainder of the lifestyle variables were also harmonious with past studies [22, 23]. Decreasing physical activity increased odds of developing fatigue. Maintaining or developing obesity or decreasing physical activity lowered odds of recovery. Developing obesity was significantly associated with increased odds of developing excessive fatigue in all but the fully adjusted analyses, but this may be explained by insufficient sample size as the analyses were adjusted for several factors.

### Health conditions

Our study supports prior findings on relationships between fatigue and depression, anxiety [19, 27], insomnia [21], and shift work disorder [8, 28]. Overall, maintaining or developing these health conditions increased odds of developing and decreased odds of recovery from excessive fatigue. Thus, depression, anxiety, insomnia, and shift work disorder seem to play clear roles in fatigue development and recovery.

Our study further indicates that changes in sleep are arguably among the most important factors, with multiple sleep changes (sleep duration, insomnia, shift work disorder) significantly increasing odds of development of excessive fatigue or lowering the odds of recovery from excessive fatigue. These findings support sleep issues as a primary mechanism for fatigue in nurses.

### Limitations

Some results were imprecise and had wide CIs due to small sample sizes within each category of predictor and outcome. Additionally, decision latitude Cronbach alphas were quite low. Therefore, these results require cautious interpretation. All measures were self-reported and collected simultaneously at each time point. Self-reported data carries the risk of inexact recall, common method bias, and cannot offer formal diagnoses of health conditions. A healthy worker effect may be at play wherein nurses who developed excessive fatigue may have dropped out or moved to day work before baseline. This may have diminished associations between work characteristics, lifestyle factors and health conditions and fatigue, potentially limiting or disguising important causal factors.

As this cohort comprises nurses and only ∼ 10% men, generalizability to non-healthcare occupations and to males is limited. However, the size and homogeneity of the cohort limit confounding by factors such as income, education, and workload. The scales we used to measure psychosocial work factors [33], alcohol consumption [39, 46], depression and anxiety [40], insomnia [42], shift work disorder [8] and fatigue [44] are validated or well-established with past epidemiological studies.

As mentioned in the analysis section, health problems associated with long sleep duration may differ from short duration. Therefore, sensitivity analyses were run on development of or recovery from excessive fatigue, excluding nurses sleeping > 8 h (Additional file 8). While the same changes in sleep duration were significant and associated in the same directions, point estimates for odds ratios differed somewhat. Notably, nurses included in the sensitivity analyses maintaining inappropriate sleep duration had an aOR of 0.12 (over 8x the odds of not recovering) compared to 0.19 (∼ 5x the odds of not recovering) and those developing inappropriate sleep duration had an aOR of 0.20 (5x the odds of not recovering) compared to 0.37 (nearly 3x the odds of not recovering). This may suggest that within this cohort short sleep duration had a somewhat stronger effect than inappropriate sleep duration on excessive fatigue, or that reduction in precision resulting from smaller sample sizes (due to removing nurses from the analyses) resulted in less accurate estimates. Overall, very few nurses in our cohort reported long sleep duration (> 8 h), this may be due to a healthy worker effect and limits our ability to assess long sleep duration’s potential impact on fatigue.

### Future research

Further research ideally would include objective measures of work characteristics, lifestyle factors, health conditions and fatigue. The implications of this study are that sleep as well as psychosocial work factors are potential targets for intervention. Emphasis in this realm should be placed on methods to improve sleep and bolster coworker support and decision latitude while reducing psychological work demands as much as possible. Workplace interventions fostering increased sleep aptitude, coworker support, decision latitude and decreased psychological work demands may be tested. Longitudinal research with more than two waves would be especially valuable to elucidate real mediating mechanisms.

## Conclusions

The results from the present study offer added assurances regarding directionality between depression, anxiety, insomnia and shift work disorder and excessive fatigue. This study underscores sleep’s important role in fatigue, and psychosocial work factors’ potential for preventing or mitigating excessive fatigue. Shift work schedules, however, seem not to play a significant role in excessive fatigue among experienced nurses.

### Electronic supplementary material

Below is the link to the electronic supplementary material.

**Additional File 1.** Work characteristics, lifestyle factors and fatigue in nurses developing excessive fatigue from 2015 to 2018.

**Additional File 2.** Work characteristics, lifestyle factors and fatigue in nurses recovering from excessive fatigue from 2015 to 2018.

**Additional File 3.** Crude and adjusted logistic regression analyses with developing excessive fatigue between baseline and follow-up as the dependent variable among Norwegians nurses.

**Additional File 4.** Crude and adjusted logistic regression analyses with recovering from excessive fatigue between baseline and follow-up as the dependent variable among Norwegians nurses.

**Additional File 5.** Additional information on analyses.

**Additional File 6.** SUSSH Questionnaire for 2015.

**Additional File 7.** SUSSH Questionnaire for 2018.

**Additional File 8.** Sensitivity analyses excluding long sleep duration.

## Data Availability

The dataset analyzed during the current study is not publicly available due to potentially sensitive and indirectly identifiable information contained within. Ethical regulations in Norway dictate that such data not be shared publicly. However, datasets are available up reasonable request from the leaders of SUSSH or the Bergen Sleep and Chronobiology Network (post@psysp.uib.no). The SUSSH web page provides contact information for SUSSH group leaders (www.uib.no/en/rg/sc/120919/survey-shift-work-sleep-and-health-sussh).

## Abbreviations

BMI: Body mass index
SUSSH: The Survey of Shift Work Sleep and Health
AUDIT-C: Alcohol Use Disorders Identification Test Consumption
OR: Odds ratio
CI: 95% confidence intervals
aOR: Adjusted odds ratio
