# Supplementary material for: Factors leading to excessive fatigue in nurses – a three-year follow-up study
Source: BMC Nurs. 2024 Jul 1;23:446. doi: 10.1186/s12912-024-02066-w (PMC11218166; doi:10.1186/s12912-024-02066-w)
Supplement: Supplementary file 5 — Additional File 5. Additional information on analyses. [file 12912_2024_2066_MOESM5_ESM.docx]

**Supplementary File 5. Additional information on analyses**

One participant had an impossible weight at baseline (likely a typo), baseline weight was therefore extrapolated based on other timepoints.

The Box-Tidwell procedure was used to assess linearity of age (the only continuous variable included in analyses) with respect to the logit for recovering from or developing excessive fatigue. A Bonferroni correction was applied in light of a three- or a five-term model. Statistical significance for Box-Tidwell was considered p <0.025 or p <0.01 respectively for crude or fully adjusted models.
