# Supplementary material for: Factors leading to excessive fatigue in nurses – a three-year follow-up study
Source: BMC Nurs. 2024 Jul 1;23:446. doi: 10.1186/s12912-024-02066-w (PMC11218166; doi:10.1186/s12912-024-02066-w)
Supplement: Supplementary file 6 — Additional File 6. SUSSH Questionnaire for 2015. [file 12912_2024_2066_MOESM6_ESM.pdf]

SPØRRESKJEMA –2015

SUSSH

Tusen takk for at du har svart på tidligere skjema fra ”Spørreundersøkelsen om skiftarbeid, søvn og helse’’! Vi gjennomfører nå en ny undersøkelse for å se hvordan det går med deltakerne over tid.

Obs! Det er viktig å besvare spørsmålene selv om du har SLUTTET som sykepleier.
Det er også viktig at du svarer selv om du IKKE er i arbeid nå, og UANSETT hvilken arbeidstidsordning du har.

Alle som sender inn utfylt skjema er med i trekningen av 25 gavekort pålydende kr. 500,-.

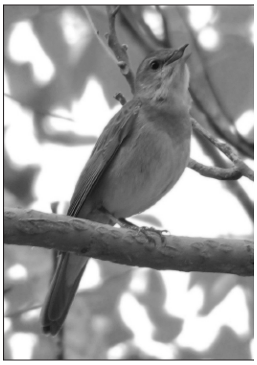

SUSSH - Nattergalen

Mange av spørsmålene omhandler forhold både i jobb og privat som kan virke inn på helsen til sykepleiere. Les spørsmålene nøye og svar med det som passer best for deg. Det finnes ikke ”rette” eller ”feile” svar. Noen spørsmål ligner på hverandre. Årsaken til dette er at spørreskjemaet er sammensatt av flere standardiserte skalaer brukt i tilsvarende forskning internasjonalt. Det er viktig at du besvarer alle spørsmålene!

Skjemaet skal leses av en maskin. Det er derfor viktig at du legger vekt på følgende ved utfyllingen:

- Bruk blå eller sort kulepenn.
- I de små avkrysningsboksene setter du et kryss inni boksen for det svaret som du mener passer best, slik: ☒ Skriver du feil, kan du ta bort krysset ved å fylle boksen helt, slik: ■ og deretter fylle ut det riktige alternativet.
- Enkelte steder skal du skrive tall.

Skriv tydelig Eksempel: | 1 | 2 | 3 | 4 | 5 | 6 | 7 | 8 | 9 | 0 |

Undersøkelsen er godkjent av Regional komité for medisinsk og helsefaglig forskningsetikk Vest-Norge, og har fått konsesjon fra Datatilsynet.

	Dag		Måned		År	
**1. Dato for utfylling**						

NÅVÆRENDE ARBEID

2. Er du i arbeid som sykepleier nå?

a. ☐ Ja ☐ Nei

b. Hvis nei, er du:

☐ I annet arbeid ☐ Student

☐ Arbeidsledig ☐ Fødselspermisjon

☐ Får arbeidsavklaringspenger ☐ Ute av jobb av annen grunn

☐ Uføretrygdet

Her kommer noen spørsmål for dem som har vært i arbeid det siste året. Dersom du ikke har vært i arbeid det siste året, gå til spørsmål 11.

3. Har du hatt sykefravær de siste 12 månedene som skyldes egen sykdom? *(Regn med syke- og egenmeldinger, men regn IKKE med fravær på grunn av sykdom hos barn/pårørende.)*

a. ☐ Ja ☐ Nei

b. Hvis ja, anslå samlet fravær de siste 12 månedene ved å sette ett kryss:

1-7 dager	8-14 dager	15-30 dager	31-90 dager	91+ dager
☐	☐	☐	☐	☐

4. a. Har du det siste året endret arbeidsplass/arbeidstidsordning? ☐ Ja ☐ Nei

Hvis ja,

b. Har du gjort dette fordi du har problemer med å takle nattarbeid? ☐ Ja ☐ Nei

c. Har du gjort dette fordi du har problemer med å takle annet turnusarbeid, som ikke inkluderer natt? ☐ Ja ☐ Nei

5. Hvor mange timer arbeider du vanligvis per uke? *(Ta med alt lønnet arbeid, inkludert overtid og eventuelt betalt studietid.)*

Eksempel: 3 7 timer 3 0 minutter

☐☐ timer ☐☐ minutter

6. Hvor mange netter har du arbeidet de siste 12 månedene? *(cirka antall)*

☐☐☐ Antall netter

7. I løpet av de siste 12 månedene, hvor mange ganger har du hatt mindre enn 11 timer fri mellom to vakter? *(cirka antall)*

☐☐☐ Antall ganger

Hvis du er i arbeid, men IKKE arbeider som sykepleier nå, vennligst gå til spørsmål 11.

8. Sett kryss ved det som best beskriver din kategori arbeidsplass *(hos hovedarbeidsgiver)***:**

☐ Somatisk sykehusavdeling/poliklinikk

☐ Psykiatrisk sykehusavdeling/poliklinikk/ambulante tjenester

☐ Sykehjem

☐ Hjemmesykepleie

☐ Helsestasjon

☐ Annet

9. Hvor stor stillingsprosent har du hos din hovedarbeidsgiver?

mindre enn 50%	50-75%	76-90%	større enn 90%
☐	☐	☐	☐

10. Sett ett kryss ved det som best beskriver din kategori arbeidstidsordning i din nåværende jobb *(hos hovedarbeidsgiver):*

- Bare dag ☐
- Bare kveld ☐
- Både dag og kveld ☐
- Bare natt ☐
- Tre-skift turnus (dag/kveld/natt) ☐
- Annen ordning som inkluderer nattarbeid ☐

SOSIALE FORHOLD

11. Er du gift, registrert partner eller samboer? ☐ Ja ☐ Nei

12. Har du barn som bor hjemme hos deg? ☐ Ja ☐ Nei

SØVN

Eksempel: 0 7 timer 3 0 minutter

13. Hvor mange timer søvn får du i gjennomsnitt per døgn? ☐☐ timer ☐☐ minutter
(Tenk deg et gjennomsnitt den siste måneden.)

14. Hvor mye søvn trenger du per døgn for å føle deg uthvilt? ☐☐ timer ☐☐ minutter

15. De 6 neste spørsmålene er knyttet til søvn og tretthet. Vær vennlig og sett kryss ved det alternativet (antall dager per uke) som passer best for deg. 0 er ingen dager i løpet av en uke, 7 er alle dager i løpet av en uke. Tenk deg et gjennomsnitt slik det har vært de siste 3 månedene:

Antall dager per uke (sett kryss)

**Hvor mange dager per uke har du:**	0	1	2	3	4	5	6	7
a. Brukt mer enn 30 minutter for å sovne inn etter at lysene ble slukket?	☐	☐	☐	☐	☐	☐	☐	☐
b. Vært våken mer enn 30 minutter innimellom søvnen?	☐	☐	☐	☐	☐	☐	☐	☐
c. Våknet mer enn 30 minutter tidligere enn du har ønsket uten å få sove igjen?	☐	☐	☐	☐	☐	☐	☐	☐
d. Følt deg for lite uthvilt etter å ha sovet?	☐	☐	☐	☐	☐	☐	☐	☐
e. Vært så søvnig/trett at det har gått ut over skole/jobb eller privatlivet?	☐	☐	☐	☐	☐	☐	☐	☐
f. Vært misfornøyd med søvnen din?	☐	☐	☐	☐	☐	☐	☐	☐

16. Har du i løpet av det siste året brukt:

a. Sovemedisin på resept ☐ Ja ☐ Nei
b. Sovemedisin uten resept ☐ Ja ☐ Nei
c. Lysbehandling ☐ Ja ☐ Nei
d. Melatonin ☐ Ja ☐ Nei

17. a. Har du en arbeidstidsordning som i perioder overlapper med tidspunkt du vanligvis sover? ☐ Ja ☐ Nei

b. Hvis ja, forårsaker dette søvnløshet og/eller forhøyet søvnighet som følge av redusert søvnmengde? ☐ Ja ☐ Nei

c. Hvis ja, har dette vart i minst 3 måneder? ☐ Ja ☐ Nei

18. **Hvor sannsynlig er det at du døser av eller sovner i følgende situasjoner, i motsetning til kun å føle deg trett? Spørsmålene gjelder din vanlige måte å reagere på i den senere tid. Selv om du ikke har gjort noe av dette i den siste tiden, så prøv likevel å finne ut hvordan situasjonene ville virke på deg. Bruk den følgende skala for å velge det mest passende tall for hver situasjon:**

**Situasjon**	ville aldri døse/sovne	en liten sjanse for å døse/sovne	moderat sjanse for å døse/sovne	stor sjanse for å døse/sovne
a. Sitte og lese	☐	☐	☐	☐
b. Se på TV	☐	☐	☐	☐
c. Sitte, inaktiv på et offentlig sted (f.eks. på teater eller et møte)	☐	☐	☐	☐
d. Som passasjer på en en-times biltur uten pause	☐	☐	☐	☐
e. Legge deg for å hvile om ettermiddagen hvis omstendighetene tillater det	☐	☐	☐	☐
f. Sitte og snakke med noen	☐	☐	☐	☐
g. Sitte stille etter lunsj (uten å ha inntatt alkohol)	☐	☐	☐	☐
h. I en bil, som har stoppet for noen få minutter i trafikken	☐	☐	☐	☐

Handlinger i søvne

	Nei	Ja, av og til	Ja, ofte	Vet ikke
19. Siste 3 måneder: Har du gått i søvne?	☐	☐	☐	☐
20. Siste 3 måneder: Har du hatt mareritt?	☐	☐	☐	☐
21. Siste 3 måneder: Har du skadet deg selv i søvne?	☐	☐	☐	☐
22. Siste 3 måneder: Har du skadet andre i søvne?	☐	☐	☐	☐
23. Siste 3 måneder: Har du utøvet seksuelle handlinger i søvne?	☐	☐	☐	☐
24. Siste 3 måneder: Har du spist mat i søvne?	☐	☐	☐	☐
25. Siste 3 måneder: Har du levd ut drømmene dine i søvne?	☐	☐	☐	☐
26. Siste 3 måneder: Har du våknet opp fra søvn i en forvirret tilstand uten å huske noe av hendelsen neste dag?	☐	☐	☐	☐
27. Siste 3 måneder: Har du våknet opp fra søvn med skrekkanfall uten å huske noe av hendelsen neste dag?	☐	☐	☐	☐

28. **Har du hatt plager (smerter, ubehag, nedsatt bevegelighet) noen gang siste 12 måneder fra:**
(Kryss av for alle kroppsdelene.)

	Aldri	Sjelden	Iblant	Ofte	Svært ofte
nakke...	☐	☐	☐	☐	☐
skulder...	☐	☐	☐	☐	☐
albue...	☐	☐	☐	☐	☐
håndledd/hender...	☐	☐	☐	☐	☐
øvre del av rygg...	☐	☐	☐	☐	☐
nedre del av rygg...	☐	☐	☐	☐	☐
hofte...	☐	☐	☐	☐	☐
kne...	☐	☐	☐	☐	☐
ankel/fot...	☐	☐	☐	☐	☐

29. **Hvor ofte drikker du alkohol?**

aldri	månedlig eller sjeldnere	to til fire ganger i måneden	to til tre ganger i uken	fire ganger i uken eller mer
☐	☐	☐	☐	☐

30. **Hvor mange alkoholenheter (en drink, ett glass vin eller en liten flaske øl) tar du på en "typisk" drikkedag?**

1-2	3-4	5-6	7-9	10 eller flere
☐	☐	☐	☐	☐

31. **Hvor ofte drikker du seks alkoholenheter eller mer på en gang?**

aldri	månedlig eller sjeldnere	noen ganger i måneden	noen ganger i uken	daglig eller nesten daglig
☐	☐	☐	☐	☐

32. Har du tidligere fått påvist…

Hjerteinfarkt	☐ Ja	☐ Nei
Hjerneslag	☐ Ja	☐ Nei

**Har du tidligere fått påvist…**			Hvis ja, får du *medikamentell* behandling for dette nå?	
Diabetes mellitus	☐ Ja	☐ Nei	☐ Ja	☐ Nei
Hypertensjon/ høyt blodtrykk	☐ Ja	☐ Nei	☐ Ja	☐ Nei
Høyt kolesterol	☐ Ja	☐ Nei	☐ Ja	☐ Nei
KOLS	☐ Ja	☐ Nei	☐ Ja	☐ Nei
Astma	☐ Ja	☐ Nei	☐ Ja	☐ Nei
Angina pectoris	☐ Ja	☐ Nei	☐ Ja	☐ Nei
Depresjon	☐ Ja	☐ Nei	☐ Ja	☐ Nei
Angst	☐ Ja	☐ Nei	☐ Ja	☐ Nei

33. Stort sett, vil du si at din helse er:

Utmerket	Meget god	God	Nokså god	Dårlig
☐	☐	☐	☐	☐

Tretthet (Fatigue)

Vi vil gjerne vite om du har følt deg sliten, svak eller i mangel av overskudd den siste måneden. Vennligst besvar ALLE spørsmålene ved å krysse av for det svaret du synes passer best for deg. Vi ønsker at du besvarer alle spørsmålene selv om du ikke har hatt slike problemer. Vi spør om hvordan du har følt deg i det siste og ikke om hvordan du følte deg for lenge siden. Hvis du har følt deg sliten lenge, ber vi om at du sammenlikner deg med hvordan du følte deg sist du var bra. *(Sett ett kryss for hver linje)*

34. Har du problemer med at du føler deg sliten?

☐ Mindre enn vanlig ☐ Ikke mer enn vanlig ☐ Mer enn vanlig ☐ Mye mer enn vanlig

35. Trenger du mer hvile?

☐ Nei, mindre enn vanlig ☐ Ikke mer enn vanlig ☐ Mer enn vanlig ☐ Mye mer enn vanlig

36. Føler du deg søvnig eller døsig?

☐ Mindre enn vanlig ☐ Ikke mer enn vanlig ☐ Mer enn vanlig ☐ Mye mer enn vanlig

37. Har du problemer med å komme i gang med ting?

☐ Mindre enn vanlig ☐ Ikke mer enn vanlig ☐ Mer enn vanlig ☐ Mye mer enn vanlig

38. Mangler du overskudd?

☐ Ikke i det hele tatt ☐ Ikke mer enn vanlig ☐ Mer enn vanlig ☐ Mye mer enn vanlig

39. Har du redusert styrke i musklene dine?

☐ Ikke i det hele tatt ☐ Ikke mer enn vanlig ☐ Mer enn vanlig ☐ Mye mer enn vanlig

40. Føler du deg svak?

☐ Mindre enn vanlig ☐ Som vanlig ☐ Mer enn vanlig ☐ Mye mer enn vanlig

41. Har du vansker med å konsentrere deg?

☐ Mindre enn vanlig ☐ Som vanlig ☐ Mer enn vanlig ☐ Mye mer enn vanlig

42. Forsnakker du deg i samtaler?

☐ Mindre enn vanlig ☐ Ikke mer enn vanlig ☐ Mer enn vanlig ☐ Mye mer enn vanlig

43. Er det vanskeligere å finne det rette ordet?

☐ Mindre enn vanlig ☐ Ikke mer enn vanlig ☐ Mer enn vanlig ☐ Mye mer enn vanlig

44. Hvordan er hukommelsen din?

☐ Bedre enn vanlig ☐ Ikke verre enn vanlig ☐ Verre enn vanlig ☐ Mye verre enn vanlig

ARBEIDSMILJØ

Here er noen spørsmål for dem som har vært i arbeid det siste året. Dersom du ikke har vært i arbeid det siste året, gå til spørsmål 78.

Positive og psykososiale faktorer i arbeidsmiljøet *(Kryss av det som passer best for alle utsagnene under.)*

	Stemmer helt	Stemmer ganske bra	Stemmer ikke særlig bra	Stemmer ikke
45. Det er rolig og behagelig stemning på min arbeidsplass.	☐	☐	☐	☐
46. Det er godt samhold.	☐	☐	☐	☐
47. Mine arbeidskamerater stiller opp for meg.	☐	☐	☐	☐
48. Det er forståelse for at jeg kan ha en dårlig dag.	☐	☐	☐	☐
49. Jeg kommer godt overens med mine overordnede.	☐	☐	☐	☐
50. Jeg trives bra med mine arbeidskamerater.	☐	☐	☐	☐

**Vedrørende ditt arbeid**	Ja, ofte	Ja, noen ganger	Nei, sjelden	Nei, så godt som aldri
51. Krever arbeidet ditt at du arbeider meget raskt?	☐	☐	☐	☐
52. Krever arbeidet ditt at du arbeider meget hardt?	☐	☐	☐	☐
53. Krever arbeidet ditt for stor arbeidsinnsats?	☐	☐	☐	☐
54. Har du tilstrekkelig tid til å utføre arbeidsoppgavene dine?	☐	☐	☐	☐
55. Forekommer det ofte motstridende krav i arbeidet ditt?	☐	☐	☐	☐
56. Får du lære nye ting i ditt arbeid?	☐	☐	☐	☐
57. Krever ditt arbeid dyktighet?	☐	☐	☐	☐
58. Krever ditt arbeid oppfinnsomhet/kreativitet?	☐	☐	☐	☐
59. Innebærer ditt arbeid at du gjør samme ting om og om igjen?	☐	☐	☐	☐
60. Har du frihet til å bestemme hvordan ditt arbeid skal utføres?	☐	☐	☐	☐
61. Har du frihet til å bestemme hva som skal utføres i ditt arbeid?	☐	☐	☐	☐

	Meget sjelden eller aldri	Nokså sjelden	Av og til	Nokså ofte	Meget ofte eller alltid
62. Er det fastsatt klare mål for din jobb?	☐	☐	☐	☐	☐
63. Vet du hva som er ditt ansvarsområde?	☐	☐	☐	☐	☐
64. Vet du nøyaktig hva som forventes av deg i jobben?	☐	☐	☐	☐	☐
65. Må du gjøre ting du mener burde vært gjort annerledes?	☐	☐	☐	☐	☐
66. Får du oppgaver uten tilstrekkelige hjelpemidler og ressurser til å fullføre dem?	☐	☐	☐	☐	☐
67. Mottar du motstridende forespørsler fra to eller flere personer?	☐	☐	☐	☐	☐
68. Fordeler din nærmeste sjef arbeidsoppgaver rettferdig og upartisk?	☐	☐	☐	☐	☐
69. Behandler din nærmeste sjef de ansatte rettferdig og upartisk?	☐	☐	☐	☐	☐
70. Er forholdet mellom deg og din nærmeste sjef en kilde til stress for deg?	☐	☐	☐	☐	☐

Her kommer noen spørsmål som retter seg mot din nærmeste leder
I hvilken grad opplever du at han eller hun:

	I liten grad				I stor grad
71. Behandler deg med respekt?	☐	☐	☐	☐	☐
72. Anerkjenner din innsats?	☐	☐	☐	☐	☐
73. Viser takknemlighet?	☐	☐	☐	☐	☐
74. Har forklart gangen i beslutningsprosessene skikkelig?	☐	☐	☐	☐	☐
75. Har gitt fornuftige og gode forklaringer på beslutningsgangen?	☐	☐	☐	☐	☐
76. Har klart å tilpasse og "skreddersy" informasjon i forhold til individuelle behov?	☐	☐	☐	☐	☐

77. Hvor ofte må du løfte mer enn 25 kilo på jobb?

0-5 ganger daglig	6-15 ganger daglig	16-30 ganger daglig	mer enn 30 ganger daglig
☐	☐	☐	☐

PSYKISK HELSE

Her kommer noen spørsmål om hvorledes du føler deg. For hvert spørsmål setter du kryss for ett av de fire svarene som best beskriver dine følelser **den siste uken**.
Ikke tenk for lenge på svaret – de spontane svarene er best.

78. Jeg føler meg nervøs og urolig
- ☐ 3 -Mesteparten av tiden
- ☐ 2 -Mye av tiden
- ☐ 1 -Fra tid til annen
- ☐ 0 -Ikke i det hele tatt

79. Jeg gleder meg fortsatt over tingene slik jeg pleide før
- ☐ 0 -Avgjort like mye
- ☐ 1 -Ikke fullt så mye
- ☐ 2 -Bare lite grann
- ☐ 3 -Ikke i det hele tatt

80. Jeg har en uroføIelse som om noe forferdelig vil skje
- ☐ 3 -Ja, og noe svært ille
- ☐ 2 -Ja, ikke så veldig ille
- ☐ 1 -Litt, bekymrer meg lite
- ☐ 0 -Ikke i det hele tatt

81. Jeg kan le og se det morsomme i situasjoner
- ☐ 0 -Like mye nå som før
- ☐ 1 -Ikke like mye nå som før
- ☐ 2 -Avgjort ikke som før
- ☐ 3 -Ikke i det hele tatt

82. Jeg har hodet fullt av bekymring
- ☐ 3 -Veldig ofte
- ☐ 2 -Ganske ofte
- ☐ 1 -Av og til
- ☐ 0 -En gang i blant

83. Jeg er i godt humør
- ☐ 3 -Aldri
- ☐ 2 -Noen ganger
- ☐ 1 -Ganske ofte
- ☐ 0 -For det meste

84. Jeg kan sitte i fred og ro og kjenne meg avslappet
- ☐ 0 -Ja, helt klart
- ☐ 1 -Vanligvis
- ☐ 2 -Ikke så ofte
- ☐ 3 -Ikke i det hele tatt

85. Jeg føler meg som om alt går langsommere
- ☐ 3 -Nesten hele tiden
- ☐ 2 -Svært ofte
- ☐ 1 -Fra tid til annen
- ☐ 0 -Ikke i det hele tatt

86. Jeg føler meg urolig som om jeg har sommerfugler i magen
- ☐ 0 -Ikke i det hele tatt
- ☐ 1 -Fra tid til annen
- ☐ 2 -Ganske ofte
- ☐ 3 -Svært ofte

87. Jeg bryr meg ikke lenger om hvordan jeg ser ut
- ☐ 3 -Ja, jeg har sluttet å bry meg
- ☐ 2 -Ikke som jeg burde
- ☐ 1 -Kan hende ikke nok
- ☐ 0 -Bryr meg som før

88. Jeg er rastløs som om jeg stadig må være aktiv
- ☐ 3 -Uten tvil svært mye
- ☐ 2 -Ganske mye
- ☐ 1 -Ikke så veldig mye
- ☐ 0 -Ikke i det hele tatt

89. Jeg ser med glede frem til hendelser og ting
- ☐ 0 -Like mye som før
- ☐ 1 -Heller mindre enn før
- ☐ 2 -Avgjort mindre enn før
- ☐ 3 -Nesten ikke i det hele tatt

90. Jeg kan plutselig få en følelse av panikk
- ☐ 3 -Uten tvil svært ofte
- ☐ 2 -Ganske ofte
- ☐ 1 -Ikke så veldig ofte
- ☐ 0 -Ikke i det hele tatt

91. Jeg kan glede meg over gode bøker, radio og TV
- ☐ 0 -Ofte
- ☐ 1 -Fra tid til annen
- ☐ 2 -Ikke så ofte
- ☐ 3 -Svært sjelden

REPRODUKSJON *(Kun for kvinner. Menn går til spørsmål 101.)*

92. Er du gravid nå? ☐ Ja ☐ Nei ☐ Usikker

93. Hvor mange barn har du født i ditt liv? Jeg har født ☐☐ barn

94. Har du det *siste året* spontanabortert (ufrivillig mistet fosteret) etter at graviditeten var sikkert påvist? ☐ Ja ☐ Nei ☐ Usikker

Hvis ja, antall ganger spontanabort det siste året: ☐☐

95. Har du fjernet livmoren? ☐ Ja ☐ Nei

96. Har du *det siste året* brukt:

P-pille (også minipille)	☐ Ja	☐ Nei
Hormonspiral	☐ Ja	☐ Nei
Vanlig spiral	☐ Ja	☐ Nei
Annen hormonbasert prevensjon (f.eks., p-sprøyte, p-ring)	☐ Ja	☐ Nei

97. Har du hatt menstruasjon siste året? ☐ Ja ☐ Nei

For de som har menstruasjon *(Svar på hva som har vært vanlig for deg den siste tiden.):*

98. Hvor lenge varer hver menstruasjon vanligvis? ☐☐ dager

99. Hvor lenge er det mellom hver menstruasjon vanligvis?
(Fra siste blødningsdag til første) ☐☐☐ dager

100. Dersom dine menstruasjoner er veldig uregelmessige, kryss her: ☐

LIVSSTIL/VEKT

101. Hvor mange kopper kaffe/te/cola (med koffeininnhold) drikker du vanligvis i løpet av en dag? Samlet antall: ☐☐ kopper

102. Røyker du daglig nå? ☐ Nei ☐ Ja Hvis ja, antall sigaretter daglig: ☐☐ sigaretter

103. Snuser du daglig nå? ☐ Nei ☐ Ja Hvis ja, antall porsjoner daglig: ☐☐ porsjoner

104. Hvordan har din fysiske aktivitet i fritiden vært det siste året? Tenk deg et ukentlig gjennomsnitt for året. Arbeidsvei regnes som fritid. Besvar begge spørsmålene ved å sette ett kryss for hver linje:

**Timer per uke:**	Ingen	Under 1 time	1-2 timer	3 timer og mer
a) Lett aktivitet (ikke svett/andpusten)	☐	☐	☐	☐
b) Hard fysisk aktivitet (svett/andpusten)	☐	☐	☐	☐

105. Hvor mye veier du? ☐☐☐ , ☐ kg

TAKK FOR AT DU HAR TATT DEG TID TIL Å DELTA I UNDERSØKELSEN

OBS! Vennligst ikke brett skjemaet! Husk å legge spørreskjemaet i svarkonvolutten.

Institutt for global helse og samfunnsmedisin
Universitetet i Bergen
Postboks 7804
5020 Bergen
siri.waage@isf.uib.no
