# Supplementary material for: Factors leading to excessive fatigue in nurses – a three-year follow-up study
Source: BMC Nurs. 2024 Jul 1;23:446. doi: 10.1186/s12912-024-02066-w (PMC11218166; doi:10.1186/s12912-024-02066-w)
Supplement: Supplementary file 7 — Additional File 7. SUSSH Questionnaire for 2018. [file 12912_2024_2066_MOESM7_ESM.pdf]

SPØRRESKJEMA – 2018

SUSSH

Tusen takk for at du har svart på tidligere skjema fra ”Spørreundersøkelsen om skiftarbeid, søvn og helse’’! Vi gjennomfører nå en ny runde for å se hvordan det går med deltakerne over tid.

Obs! Det er viktig å besvare spørsmålene selv om du har SLUTTET som sykepleier.
Det er også viktig at du svarer selv om du IKKE er i arbeid nå, og UANSETT hvilken arbeidstidsordning du har.

Alle som sender inn utfylt skjema er med i trekningen av 25 gavekort pålydende kr. 500,-.

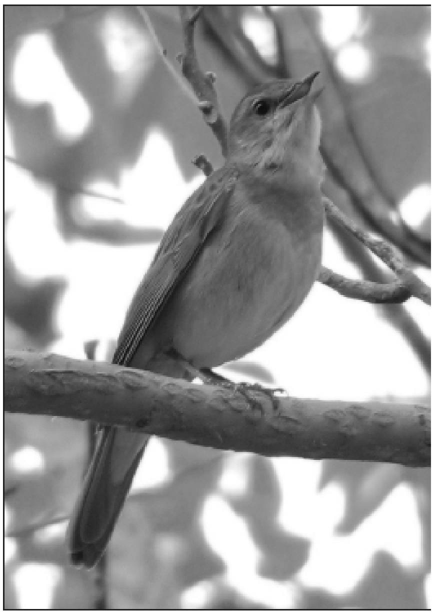

Mange av spørsmålene omhandler forhold både i jobb og privat som kan virke inn på helsen til sykepleiere. Les spørsmålene nøye og svar med det som passer best for deg. Det finnes ikke ”rette” eller ”feile” svar. Noen spørsmål ligner på hverandre. Årsaken til dette er at spørreskjemaet er sammensatt av flere standardiserte skalaer brukt i tilsvarende forskning internasjonalt. Det er viktig at du besvarer alle spørsmålene!

Skjemaet skal leses av en maskin. Det er derfor viktig at du legger vekt på følgende ved utfyllingen:

- Bruk blå eller sort kulepenn.
- I de små avkrysningsboksene setter du et kryss inni boksen for det svaret som du mener passer best, slik: ☒ Skriver du feil, kan du ta bort krysset ved å fylle boksen helt, slik: ■ og deretter fylle ut det riktige alternativet.
- Enkelte steder kan du skrive tall.

Skriv tydelig Eksempel: 1 2 3 4 5 6 7 8 9 0

Undersøkelsen er godkjent av Regional komité for medisinsk og helsefaglig forskningsetikk Vest-Norge, og har fått konsesjon fra Datatilsynet.

Resultater av forskning fra tidligere runder av spørreundersøkelsen SUSSH kan du lese mer om på **www.sussh.no**

Dag		Måned		År	

1. Dato for utfylling ☐☐☐☐☐☐

NÅVÆRENDE ARBEID

2. Er du i arbeid som sykepleier nå?

a. ☐ Ja ☐ Nei

b. Hvis nei, er du:

☐ I annet arbeid	☐ Student
☐ Arbeidsledig	☐ Fødselspermisjon
☐ Får arbeidsavklaringspenger	☐ Ute av jobb av annen grunn
☐ Uføretrygdet	

Her er noen spørsmål for dem som har vært i arbeid det siste året. Dersom du ikke har vært i arbeid det siste året, gå til spørsmål 11.

3. Har du hatt sykefravær de siste 12 månedene som skyldes egen sykdom? *(Regn med syke- og egenmeldinger, men regn IKKE med fravær på grunn av sykdom hos barn/pårørende.)*

a. ☐ Ja ☐ Nei

b. Hvis ja, anslå samlet fravær de siste 12 månedene ved å sette ett kryss:

1-7 dager	8-14 dager	15-30 dager	31-90 dager	91+ dager
☐	☐	☐	☐	☐

4.

a.	Har du det siste året endret arbeidsplass/arbeidstidsordning?	☐ Ja	☐ Nei
	Hvis ja,		
b.	Har du gjort dette fordi du har problemer med å takle nattarbeid?	☐ Ja	☐ Nei
c.	Har du gjort dette fordi du har problemer med å takle annet turnusarbeid, som ikke inkluderer natt?	☐ Ja	☐ Nei

Eksempel: 3 7 timer 3 0 minutter

5. Hvor mange timer arbeider du vanligvis per uke?
(Ta med alt lønnet arbeid, inkludert overtid og eventuelt betalt studietid.) ☐☐ timer ☐☐ minutter

6. Hvor mange netter har du arbeidet de siste 12 månedene?
(cirka antall) ☐☐☐ Antall netter

7. I løpet av de siste 12 månedene, hvor mange ganger har du hatt mindre enn 11 timer fri mellom to vakter? *(cirka antall)* ☐☐☐ Antall ganger

Hvis du er i arbeid, men IKKE arbeider som sykepleier nå, vennligst gå til spørsmål 11.

8. Sett kryss ved det som best beskriver din kategori arbeidsplass *(hos hovedarbeidsgiver)*:

☐ Somatisk sykehusavdeling/poliklinikk
☐ Psykiatrisk sykehusavdeling/poliklinikk/ambulante tjenester
☐ Sykehjem
☐ Hjemmesykepleie
☐ Helsestasjon
☐ Annet

9. **Hvor stor stillingsprosent har du hos din hovedarbeidsgiver?**

Mindre enn 50%	50-75%	76-90%	Mer enn 90%
☐	☐	☐	☐

10. **Sett ett kryss ved det som best beskriver din kategori <u>arbeidstidsordning</u> i din nåværende jobb** *<u>(hos hovedarbeidsgiver):</u>*

- ☐ Bare dag
- ☐ Bare kveld
- ☐ Både dag og kveld
- ☐ Bare natt
- ☐ Tre-skift turnus (dag/kveld/natt)
- ☐ Annen ordning som inkluderer nattarbeid

SOSIALE FORHOLD

11. **Er du gift, registrert partner eller samboer?**	☐ Ja	☐ Nei
12. **Har du barn under 2 år som bor hjemme hos deg?**	☐ Ja	☐ Nei
13. **Har du barn som er 2 år eller eldre som bor hjemme hos deg?**	☐ Ja	☐ Nei

SØVN

Eksempel: 0 7 timer 3 0 minutter

14. **Hvor mange timer søvn får du i gjennomsnitt per døgn?** ☐☐ timer ☐☐ minutter
(Tenk deg et gjennomsnitt den siste måneden.)

15. **Hvor mye søvn trenger du per døgn for å føle deg uthvilt?** ☐☐ timer ☐☐ minutter

16. **De 6 neste spørsmålene er knyttet til søvn og tretthet. Vær vennlig og sett kryss ved det alternativet (antall dager per uke) som passer best for deg. 0 er ingen dager i løpet av en uke, 7 er alle dager i løpet av en uke. Tenk deg et gjennomsnitt slik det har vært de siste 3 månedene:**

Antall dager per uke (sett kryss)

**Hvor mange dager per uke har du:**	0	1	2	3	4	5	6	7
a. Brukt mer enn 30 minutter for å sovne etter at lysene ble slukket?	☐	☐	☐	☐	☐	☐	☐	☐
b. Vært våken mer enn 30 minutter innimellom søvnen?	☐	☐	☐	☐	☐	☐	☐	☐
c. Våknet mer enn 30 minutter tidligere enn du har ønsket uten å få sove igjen?	☐	☐	☐	☐	☐	☐	☐	☐
d. Følt deg for lite uthvilt etter å ha sovet?	☐	☐	☐	☐	☐	☐	☐	☐
e. Vært så søvnig/trett at det har gått ut over skole/jobb eller privatlivet?	☐	☐	☐	☐	☐	☐	☐	☐
f. Vært misfornøyd med søvnen din?	☐	☐	☐	☐	☐	☐	☐	☐

17. **Har du i løpet av det siste året brukt:**

a. Sovemedisin på resept	☐ Ja	☐ Nei
b. Sovemedisin uten resept	☐ Ja	☐ Nei
c. Lysbehandling	☐ Ja	☐ Nei
d. Melatonin	☐ Ja	☐ Nei

18. a. Har du en arbeidstidsordning som i perioder overlapper med tidspunkt du vanligvis sover? ☐ Ja ☐ Nei

b. Hvis ja, forårsaker dette søvnløshet og/eller forhøyet søvnighet som følge av redusert søvnmengde? ☐ Ja ☐ Nei

c. Hvis ja, har dette vart i minst 3 måneder? ☐ Ja ☐ Nei

d. Hvis ja, påvirker søvn- eller tretthetsproblemet dine sosiale, familiære eller arbeidsforhold negativt?

Ikke i det hele tatt	Litt	Noe	Mye	Veldig mye
☐	☐	☐	☐	☐

19. Hvor sannsynlig er det at du døser av eller sovner i følgende situasjoner, i motsetning til kun å føle deg trett? Spørsmålene gjelder din vanlige måte å reagere på i den senere tid. Selv om du ikke har gjort noe av dette i den siste tiden, så prøv likevel å finne ut hvordan situasjonene ville virke på deg. Bruk den følgende skala for å velge det som passer for hver situasjon:

**Situasjon**	ville aldri døse/sovne	en liten sjanse for å døse/sovne	moderat sjanse for å døse/sovne	stor sjanse for å døse/sovne
a. Sitte og lese	☐	☐	☐	☐
b. Se på TV	☐	☐	☐	☐
c. Sitte, inaktiv på et offentlig sted (f.eks. på teater eller et møte)	☐	☐	☐	☐
d. Som passasjer på en en-times biltur uten pause	☐	☐	☐	☐
e. Legge deg for å hvile om ettermiddagen hvis omstendighetene tillater det	☐	☐	☐	☐
f. Sitte og snakke med noen	☐	☐	☐	☐
g. Sitte stille etter lunsj (uten å ha inntatt alkohol)	☐	☐	☐	☐
h. I en bil, som har stoppet for noen få minutter i trafikken	☐	☐	☐	☐

HELSE

20. Har du tidligere fått påvist…

Hjerteinfarkt	☐ Ja	☐ Nei	Angst	☐ Ja	☐ Nei
Hjerneslag	☐ Ja	☐ Nei	Multippel Sklerose (MS)	☐ Ja	☐ Nei
Diabetes mellitus	☐ Ja	☐ Nei	Reflukssykdom	☐ Ja	☐ Nei
Hypertensjon/ høyt blodtrykk	☐ Ja	☐ Nei	Irritabel tarm	☐ Ja	☐ Nei
Høyt kolesterol	☐ Ja	☐ Nei	Tykktarmskreft	☐ Ja	☐ Nei
KOLS	☐ Ja	☐ Nei	Brystkreft	☐ Ja	☐ Nei
Astma	☐ Ja	☐ Nei	Hypertyreose/høyt stoffskifte	☐ Ja	☐ Nei
Angina pectoris	☐ Ja	☐ Nei	Hypotyreose/lavt stoffskifte	☐ Ja	☐ Nei
Depresjon	☐ Ja	☐ Nei			

21. Har du hatt infeksjoner i løpet av de siste 3 måneder (Kryss av for alle infeksjoner som passer):

	0 ganger	1 gang	2 ganger	3 ganger	Mer enn 3 ganger
Forkjølelse	☐	☐	☐	☐	☐
Lungebetennelse/bronkitt	☐	☐	☐	☐	☐
Bihulebetennelse	☐	☐	☐	☐	☐
Mage/tarminfeksjon med oppkast eller diare	☐	☐	☐	☐	☐
Urinveisinfeksjon	☐	☐	☐	☐	☐
Andre infeksjoner	☐	☐	☐	☐	☐

22. Nedenfor følger spørsmål om plager i forskjellige kroppsdeler.
Kryss av for symptomer og plager du eventuelt har hatt i løpet av de SISTE 4 UKER.
Sett ett kryss under INTENSITET og eventuelt ett under VARIGHET for hvert av spørsmålene.

	**Plagenes intensitet**				**Ved plager: Varighet tilsammen**			
**Marker ved å sette en X i rutene for det som passer for deg**	Ikke plaget	Litt plaget	Ganske plaget	Svært plaget	1-5 dager	6-10 dager	11-14 dager	15-28 dager
Smerter i nakken, skuldre eller øvre del av ryggen	☐	☐	☐	☐	☐	☐	☐	☐
Smerter i nedre del av ryggen	☐	☐	☐	☐	☐	☐	☐	☐
Smerter i armer, håndledd eller hender	☐	☐	☐	☐	☐	☐	☐	☐
Smerter i hofter, ben, knær eller føtter	☐	☐	☐	☐	☐	☐	☐	☐
Hodepine eller migrene	☐	☐	☐	☐	☐	☐	☐	☐
Smerter i magen	☐	☐	☐	☐	☐	☐	☐	☐

Tretthet (Fatigue)

Vi vil gjerne vite om du har følt deg sliten, svak eller i mangel av overskudd den siste måneden. Vennligst besvar ALLE spørsmålene ved å krysse av for det svaret du synes passer best for deg. Vi ønsker at du besvarer alle spørsmålene selv om du ikke har hatt slike problemer. Vi spør om hvordan du har følt deg i det siste og ikke om hvordan du følte deg for lenge siden. Hvis du har følt deg sliten lenge, ber vi om at du sammenlikner deg med hvordan du følte deg sist du var bra. *(Sett ett kryss for hver linje)*

23. Har du problemer med at du føler deg sliten?

☐ Mindre enn vanlig ☐ Ikke mer enn vanlig ☐ Mer enn vanlig ☐ Mye mer enn vanlig

24. Trenger du mer hvile?

☐ Nei, mindre enn vanlig ☐ Ikke mer enn vanlig ☐ Mer enn vanlig ☐ Mye mer enn vanlig

25. Føler du deg søvnig eller døsig?

☐ Mindre enn vanlig ☐ Ikke mer enn vanlig ☐ Mer enn vanlig ☐ Mye mer enn vanlig

26. Har du problemer med å komme i gang med ting?

☐ Mindre enn vanlig ☐ Ikke mer enn vanlig ☐ Mer enn vanlig ☐ Mye mer enn vanlig

27. Mangler du overskudd?

☐ Ikke i det hele tatt ☐ Ikke mer enn vanlig ☐ Mer enn vanlig ☐ Mye mer enn vanlig

28. Har du redusert styrke i musklene dine?

☐ Ikke i det hele tatt ☐ Ikke mer enn vanlig ☐ Mer enn vanlig ☐ Mye mer enn vanlig

29. Føler du deg svak?

☐ Mindre enn vanlig ☐ Som vanlig ☐ Mer enn vanlig ☐ Mye mer enn vanlig

30. Har du vansker med å konsentrere deg?

☐ Mindre enn vanlig ☐ Som vanlig ☐ Mer enn vanlig ☐ Mye mer enn vanlig

31. Forsnakker du deg i samtaler?

☐ Mindre enn vanlig ☐ Ikke mer enn vanlig ☐ Mer enn vanlig ☐ Mye mer enn vanlig

32. Er det vanskeligere å finne det rette ordet?

☐ Mindre enn vanlig ☐ Ikke mer enn vanlig ☐ Mer enn vanlig ☐ Mye mer enn vanlig

33. Hvordan er hukommelsen din?

☐ Bedre enn vanlig ☐ Ikke verre enn vanlig ☐ Verre enn vanlig ☐ Mye verre enn vanlig

34. Hvis du føler deg sliten for tiden, omtrent hvor lenge har det vart? (Ett eller ingen kryss)

☐ Mindre enn en uke ☐ Mindre enn tre måneder ☐ Mellom tre og seks måneder ☐ Seks måneder eller mer

35. Stort sett, vil du si at din helse er:

Utmerket	Meget god	God	Nokså god	Dårlig
☐	☐	☐	☐	☐

36. Har du magesmerter minst en dag i uken? ☐ Nei ☐ Ja ☐ Vet ikke

I så fall hvor lenge har du hatt dem?

Under 1 mnd	1-3 mnd	3-6 mnd	Over 6 mnd
☐	☐	☐	☐

37. Hvis du har magesmerter minst en dag i uken, har du i minst ett av tre tilfeller disse smertene i forbindelse med at:

a. ... du har avføring, eller like før eller like etter avføring?	☐ Nei	☐ Ja	☐ Vet ikke
b. ... avføringen er løsere eller hardere enn vanlig?	☐ Nei	☐ Ja	☐ Vet ikke
c. ... avføringen kommer hyppigere eller sjeldnere enn vanlig?	☐ Nei	☐ Ja	☐ Vet ikke

ARBEIDSMILJØ

Her er noen spørsmål for dem som har vært i arbeid det siste året. Dersom du ikke har vært i arbeid det siste året, gå til spørsmål 56. *(Kryss av det som passer best for alle utsagnene under.)*

	Stemmer helt	Stemmer ganske bra	Stemmer ikke særlig bra	Stemmer ikke
38. Det er rolig og behagelig stemning på min arbeidsplass	☐	☐	☐	☐
39. Det er godt samhold	☐	☐	☐	☐
40. Mine arbeidskamerater stiller opp for meg	☐	☐	☐	☐
41. Det er forståelse for at jeg kan ha en dårlig dag	☐	☐	☐	☐
42. Jeg kommer godt overens med mine overordnede	☐	☐	☐	☐
43. Jeg trives bra med mine arbeidskamerater	☐	☐	☐	☐

Vedrørende ditt arbeid *(Kryss av på alle spørsmålene under.)*

	Ja, ofte	Ja, noen ganger	Nei, sjelden	Nei, så godt som aldri
44. Krever arbeidet ditt at du arbeider meget raskt?	☐	☐	☐	☐
45. Krever arbeidet ditt at du arbeider meget hardt?	☐	☐	☐	☐
46. Krever arbeidet ditt for stor arbeidsinnsats?	☐	☐	☐	☐
47. Har du tilstrekkelig tid til å utføre arbeidsoppgavene dine?	☐	☐	☐	☐
48. Forekommer det ofte motstridende krav i arbeidet ditt?	☐	☐	☐	☐
49. Får du lære nye ting i ditt arbeid?	☐	☐	☐	☐
50. Krever ditt arbeid dyktighet?	☐	☐	☐	☐
51. Krever ditt arbeid oppfinnsomhet/kreativitet?	☐	☐	☐	☐
52. Innebærer ditt arbeid at du gjør samme ting om og om igjen?	☐	☐	☐	☐
53. Har du frihet til å bestemme hvordan ditt arbeid skal utføres?	☐	☐	☐	☐
54. Har du frihet til å bestemme hva som skal utføres i ditt arbeid?	☐	☐	☐	☐

ARBEIDSRELATERTE HENDELSER

55. Hvor mange...

a. .... ganger har du opplevd å ufrivillig døse av på jobb den siste måneden? ☐☐☐

b. .... ganger har du opplevd å døse av/sovne mens du selv har kjørt bil til eller fra jobben det siste året? ☐☐☐

c. .... arbeidsrelaterte ulykker/uhell som har ført til skader på deg selv, har du følt at du har hatt skyld i det siste året? ☐☐☐

d. .... arbeidsrelaterte nesten-ulykker/nesten-uhell som kunne ha ført til skader på deg selv, har du følt at du har hatt skyld i det siste året? ☐☐☐

e. .... arbeidsrelaterte ulykker/uhell som har ført til skader på pasienter/andre, har du følt at du har hatt skyld i det siste året? ☐☐☐

f. .... arbeidsrelaterte nesten-ulykker/nesten-uhell som kunne ha ført til skader på pasienter/andre, har du følt at du har hatt skyld i det siste året? ☐☐☐

g. .... arbeidsrelaterte ulykker/uhell som har ført til skade på utstyr, har du følt at du har hatt skyld i det siste året? ☐☐☐

h. .... arbeidsrelaterte nesten-ulykker/nesten-uhell som kunne ha ført til skade på utstyr, har du følt at du har hatt skyld i det siste året? ☐☐☐

56. Nedenfor er det eksempler på hva som kan skje med folk i hverdagen. Noe kan skje ofte og noe sjeldent. Vi vil vite hvor ofte, i gjennomsnitt, du mener hver av tingene under har skjedd med deg i løpet av siste måned. Sett kryss i den ruten som best beskriver hvor ofte tingene har skjedd med deg.

	En gang eller sjeldnere siste måned	Mer enn en gang siste måned, men sjeldnere enn en gang i uken	Omtrent en gang i uken	Oftere enn en gang i uken men sjeldnere enn en gang per dag	En gang per dag eller oftere
Måtte sjekke at du har gjort noe som du skulle gjort.	☐	☐	☐	☐	☐
Glemt tidspunktet for når noe skjedde, for eksempel om det var i går eller forrige uke.	☐	☐	☐	☐	☐
Glemt noe du ble fortalt dagen før eller noen få dager siden, og kanskje måtte bli minnet om det.	☐	☐	☐	☐	☐
Begynt å lese noe (en bok eller avisartikkel, eller et blad) uten å innse at du har lest det før.	☐	☐	☐	☐	☐
Opplevd at du har et «ord på tungen». Du vet hva det er, men kan ikke helt komme på det.	☐	☐	☐	☐	☐
Fullstendig glemt å gjøre ting du sa du skulle gjøre og ting du planla å gjøre.	☐	☐	☐	☐	☐
Glemt viktige detaljer om noe du har gjort eller hva som skjedde deg dagen før.	☐	☐	☐	☐	☐
Glemt hva du nettopp sa mens du snakket med noen og kanskje sagt «hva snakket jeg om?»	☐	☐	☐	☐	☐
Ikke klart å følge den røde tråden i en historie når du leser en avis eller et blad, ikke holdt styr på hva det handler om.	☐	☐	☐	☐	☐
Glemt å fortelle noen noe viktig, kanskje glemt å formidle en viktig beskjed eller minne noen om noe.	☐	☐	☐	☐	☐
Blandet sammen detaljer om hva noen fortalte deg.	☐	☐	☐	☐	☐
Glemt hvor ting vanligvis er eller lett etter dem på feilt sted.	☐	☐	☐	☐	☐
Gjentatt noe du nettopp fortalte til noen eller stilt det samme spørsmålet to ganger.	☐	☐	☐	☐	☐

PSYKISK HELSE

Her kommer noen spørsmål om hvorledes du føler deg. For hvert spørsmål setter du kryss for ett av de fire svarene som best beskriver dine følelser **den siste uken**.
Ikke tenk for lenge på svaret – de spontane svarene er best.

57. Jeg føler meg nervøs og urolig
- ☐ 3 -Mesteparten av tiden
- ☐ 2 -Mye av tiden
- ☐ 1 -Fra tid til annen
- ☐ 0 -Ikke i det hele tatt

58. Jeg gleder meg fortsatt over tingene slik jeg pleide før
- ☐ 0 -Avgjort like mye
- ☐ 1 -Ikke fullt så mye
- ☐ 2 -Bare lite grann
- ☐ 3 -Ikke i det hele tatt

59. Jeg har en urofølelse som om noe forferdelig vil skje
- ☐ 3 -Ja, og noe svært ille
- ☐ 2 -Ja, ikke så veldig ille
- ☐ 1 -Litt, bekymrer meg lite
- ☐ 0 -Ikke i det hele tatt

60. Jeg kan le og se det morsomme i situasjoner
- ☐ 0 -Like mye nå som før
- ☐ 1 -Ikke like mye nå som før
- ☐ 2 -Avgjort ikke som før
- ☐ 3 -Ikke i det hele tatt

61. Jeg har hodet fullt av bekymring
- ☐ 3 -Veldig ofte
- ☐ 2 -Ganske ofte
- ☐ 1 -Av og til
- ☐ 0 -En gang i blant

62. Jeg er i godt humør
- ☐ 3 -Aldri
- ☐ 2 -Noen ganger
- ☐ 1 -Ganske ofte
- ☐ 0 -For det meste

63. Jeg kan sitte i fred og ro og kjenne meg avslappet
- ☐ 0 -Ja, helt klart
- ☐ 1 -Vanligvis
- ☐ 2 -Ikke så ofte
- ☐ 3 -Ikke i det hele tatt

64. Jeg føler meg som om alt går langsommere
- ☐ 3 -Nesten hele tiden
- ☐ 2 -Svært ofte
- ☐ 1 -Fra tid til annen
- ☐ 0 -Ikke i det hele tatt

65. Jeg føler meg urolig som om jeg har sommerfugler i magen
- ☐ 0 -Ikke i det hele tatt
- ☐ 1 -Fra tid til annen
- ☐ 2 -Ganske ofte
- ☐ 3 -Svært ofte

66. Jeg bryr meg ikke lenger om hvordan jeg ser ut
- ☐ 3 -Ja, jeg har sluttet å bry meg
- ☐ 2 -Ikke som jeg burde
- ☐ 1 -Kan hende ikke nok
- ☐ 0 -Bryr meg som før

67. Jeg er rastløs som om jeg stadig må være aktiv
- ☐ 3 -Uten tvil svært mye
- ☐ 2 -Ganske mye
- ☐ 1 -Ikke så veldig mye
- ☐ 0 -Ikke i det hele tatt

68. Jeg ser med glede frem til hendelser og ting
- ☐ 0 -Like mye som før
- ☐ 1 -Heller mindre enn før
- ☐ 2 -Avgjort mindre enn før
- ☐ 3 -Nesten ikke i det hele tatt

69. Jeg kan plutselig få en følelse av panikk
- ☐ 3 -Uten tvil svært ofte
- ☐ 2 -Ganske ofte
- ☐ 1 -Ikke så veldig ofte
- ☐ 0 -Ikke i det hele tatt

70. Jeg kan glede meg over gode bøker, radio og TV
- ☐ 0 -Ofte
- ☐ 1 -Fra tid til annen
- ☐ 2 -Ikke så ofte
- ☐ 3 -Svært sjelden

REPRODUKSJON *(Kun for kvinner. Menn går til spørsmål 80.)*

71. Er du gravid nå? ☐ Ja ☐ Nei ☐ Usikker

72. Hvor mange barn har du født i ditt liv? Jeg har født ☐☐ barn

73. Har du det *siste året* spontanabortert (ufrivillig mistet fosteret) etter at graviditeten var sikkert påvist? ☐ Ja ☐ Nei ☐ Usikker

Hvis ja, antall ganger spontanabort det siste året: ☐☐

74. Har du fjernet livmoren? ☐ Ja ☐ Nei

75. Har du *det siste året* brukt:

P-pille (også minipille)	☐ Ja	☐ Nei
Hormonspiral	☐ Ja	☐ Nei
Vanlig spiral	☐ Ja	☐ Nei
Annen hormonbasert prevensjon (f.eks., p-sprøyte, p-ring)	☐ Ja	☐ Nei

76. Har du hatt menstruasjon siste året? ☐ Ja ☐ Nei

For de som har menstruasjon *(Svar på hva som har vært vanlig for deg den siste tiden.):*

77. Hvor lenge varer hver menstruasjon vanligvis? ☐☐ dager

78. Hvor lenge er det mellom hver menstruasjon vanligvis?
(Fra siste blødningsdag til første) ☐☐☐ dager

79. Dersom dine menstruasjoner er veldig uregelmessige, kryss her: ☐

LIVSSTIL/VEKT

80. Hvor mange kopper kaffe/te/cola (med koffeininnhold) drikker du vanligvis i løpet av en dag? Samlet antall: ☐☐ kopper

81. Røyker du daglig nå? ☐ Nei ☐ Ja Hvis ja, antall sigaretter daglig: ☐☐ sigaretter

82. Snuser du daglig nå? ☐ Nei ☐ Ja Hvis ja, antall porsjoner daglig: ☐☐ porsjoner

83. Hvordan har din fysiske aktivitet i fritiden vært det siste året? Tenk deg et ukentlig gjennomsnitt for året. Arbeidsvei regnes som fritid. Besvar begge spørsmålene ved å sette ett kryss for hver linje:

**Timer per uke:**	Ingen	Under 1 time	1-2 timer	3 timer og mer
a) Lett aktivitet (ikke svett/andpusten)	☐	☐	☐	☐
b) Hard fysisk aktivitet (svett/andpusten)	☐	☐	☐	☐

84. Hvor mye veier du? ☐☐☐,☐ kg

85. Hvor ofte drikker du alkohol?

Aldri	Månedlig eller sjeldnere	To til fire ganger i måneden	To til tre ganger i uken	Fire ganger i uken eller mer
☐	☐	☐	☐	☐

86. Hvor mange alkoholenheter (en drink, ett glass vin eller en liten flaske øl) tar du på en "typisk" drikkedag?

1-2	3-4	5-6	7-9	10 eller flere
☐	☐	☐	☐	☐

87. Hvor ofte drikker du seks alkoholenheter eller mer på en gang?

Aldri	Månedlig eller sjeldnere	Noen ganger i måneden	Noen ganger i uken	Daglig eller nesten daglig
☐	☐	☐	☐	☐

88. Er du i ditt nåværende arbeid som sykepleier i kontakt med følgende:

	Daglig	Hver uke	Hver måned	Sjeldnere enn hver måned	Aldri
Anestesigasser	☐	☐	☐	☐	☐
Røntgenstråler	☐	☐	☐	☐	☐
Cytostatica	☐	☐	☐	☐	☐
Felt fra MR-maskin	☐	☐	☐	☐	☐

89. Hvor ofte må du løfte mer enn 25 kilo på jobb?

0-5 ganger daglig	6-15 ganger daglig	16-30 ganger daglig	mer enn 30 ganger daglig
☐	☐	☐	☐

TAKK FOR AT DU HAR TATT DEG TID TIL Å DELTA I UNDERSØKELSEN

SUSSH har nå pågått i 10 år og vi vil takke alle som har deltatt. Vi har fått belyst viktige sider ved arbeidssituasjonen til sykepleiere. Nåværende konsesjonstid for undersøkelsen utløper snart. Vi vurderer å søke etisk komite om å fortsette. Dere vil i så fall bli invitert til å delta i nye runder med spørreskjema.

OBS! Vennligst ikke brett skjemaet! Husk å legge spørreskjemaet i svarkonvolutten.

Institutt for global helse og samfunnsmedisin
Universitetet i Bergen
Postboks 7804
5020 Bergen
siri.waage@uib.no
